# Large-area synthesis of nanoscopic catalyst-decorated conductive MOF film using microfluidic-based solution shearing

**DOI:** 10.1038/s41467-021-24571-1

**Published:** 2021-07-13

**Authors:** Jin-Oh Kim, Won-Tae Koo, Hanul Kim, Chungseong Park, Taehoon Lee, Calvin Andreas Hutomo, Siyoung Q. Choi, Dong Soo Kim, Il-Doo Kim, Steve Park

**Affiliations:** 1 Department of Materials Science and Engineering, Korea Advanced Institute of Science and Technology (KAIST), Daejeon, Republic of Korea; 2 Membrane Innovation Center for Anti-virus & Air-quality Control, KAIST Institute for Nanocentury, Daejeon, Republic of Korea; 3 Department of Chemical and Biomolecular Engineering, Korea Advanced Institute of Science and Technology (KAIST), Daejeon, Korea; 4 Department of Creative Convergence Engineering, Hanbat National University, Daejeon, Korea

**Keywords:** Sensors and biosensors, Organic-inorganic nanostructures, Design, synthesis and processing

## Abstract

Conductive metal-organic framework (C-MOF) thin-films have a wide variety of potential applications in the field of electronics, sensors, and energy devices. The immobilization of various functional species within the pores of C-MOFs can further improve the performance and extend the potential applications of C-MOFs thin films. However, developing facile and scalable synthesis of high quality ultra-thin C-MOFs while simultaneously immobilizing functional species within the MOF pores remains challenging. Here, we develop microfluidic channel-embedded solution-shearing (MiCS) for ultra-fast (≤5 mm/s) and large-area synthesis of high quality nanocatalyst-embedded C-MOF thin films with thickness controllability down to tens of nanometers. The MiCS method synthesizes nanoscopic catalyst-embedded C-MOF particles within the microfluidic channels, and simultaneously grows catalyst-embedded C-MOF thin-film uniformly over a large area using solution shearing. The thin film displays high nitrogen dioxide (NO_2_) sensing properties at room temperature in air amongst two-dimensional materials, owing to the high surface area and porosity of the ultra-thin C-MOFs, and the catalytic activity of the nanoscopic catalysts embedded in the C-MOFs. Therefore, our method, *i.e*. MiCS, can provide an efficient way to fabricate highly active and conductive porous materials for various applications.

## Abstract (web summary)

The immobilization of catalysts within the pores of conductive metal-organic frameworks (C-MOFs) via facile and scalable methodologies remains challenging. Here the authors report a microfluidic channel-embedded solution shearing process that enables the high throughput, large-area, single-step preparation of Pt nanocatalyst-embedded C-MOF thin films.

## Introduction

Conductive metal–organic frameworks (C-MOFs) are emerging materials receiving a great deal of interest in recent years due to their many attractive properties, such as high porosity, narrow pore-size distribution and periodically organized pores, adjustable bandgap, and designable electrical charge transport properties^1–5^. These properties broaden the applicability of metal–organic frameworks (MOFs) (which have traditionally been electrically insulating) to various applications such as transistors^6^, electrodes^7–10^, and resistive chemical sensors^11–14^.

The key features of MOFs are their high porosity and regularly arranged pores. These pores can be utilized to immobilize nanoscale catalysts such as Au, Pd, and Pt, and since the reactivity of the catalysts improves with increasing surface area, well-dispersed nanoscale catalysts can drastically enhance the overall catalytic performance of MOFs^15–19^. Recently, the immobilization of nanocatalyst has been applied to C-MOFs, and was demonstrated of enhancing the performance of Li-S batteries^20^ and gas sensors^15^.

The C-MOF applications mentioned above require the formation of high-quality thin films with controlled film thickness down to nanometer scale (<100 nm), smooth and uniform surface, and densely packed thin-film MOF particles to ensure fast transport of charge carriers across the thin film^21^. Furthermore, immobilization of nanocatalyst into the pores is needed to optimize the catalytic performance of C-MOF thin films. Currently, the main techniques to generate nanoscale thin films of C-MOFs are layer-by-layer^14,22^, and interfacial synthesis^6,23,24^. Although high-quality C-MOF films can be generated with these techniques, simultaneous immobilization of nanocatalyst during C-MOF thin-film synthesis is difficult. In general, embedding of nanocatalyst within MOF films has been done by the following methods;^25,26^ (1) synthesis of metal nanoparticles (NPs) in (or on) pre-synthesized MOFs, (2) assembly of MOFs around pre-synthesized metal NPs, and (3) mixing all precursors and simultaneous growth of MOFs and metal NPs (in situ growth). The nanocatalyst-embedded MOF crystals are then deposited as a thin film^11,12,27^. However, these techniques inevitably require multiple relatively slow processes and often result in poor thin-film quality. To the best of our knowledge, there is currently no technique to generate high-quality nanoscale C-MOF thin-film with embedded nanocatalyst particles within the pores.

Herein, we introduce microfluidic channel-embedded solution shearing (MiCS) as a means to immobilize metal NPs (i.e., nanocatalysts) into the C-MOFs pores during thin-film synthesis, through which high-quality nanocatalyst-embedded C-MOF thin films are generated with thickness control down to tens of nanometers. Furthermore, contrary to other MOF thin-film growth techniques^14,22^, MiCS can generate MOF thin film over a large area in a scalable and high-throughput (5 mm/s) manner, rendering it a feasible technique for large-scale manufacturing. Solution shearing is a technique analogous to blade coating, where a fixed amount of solution is sandwiched between a moving blade and a heated substrate^28–30^. A meniscus (curved air–liquid interface) naturally forms between the blade and the substrate, and as the meniscus moves along with the moving blade, a thin film is deposited across the substrate as solution-to-solid transition occurs near the edge of the meniscus^28^.

## Results

### Preparation of nanocatalyst-embedded C-MOF thin films using MiCS

**Fig. 1:**
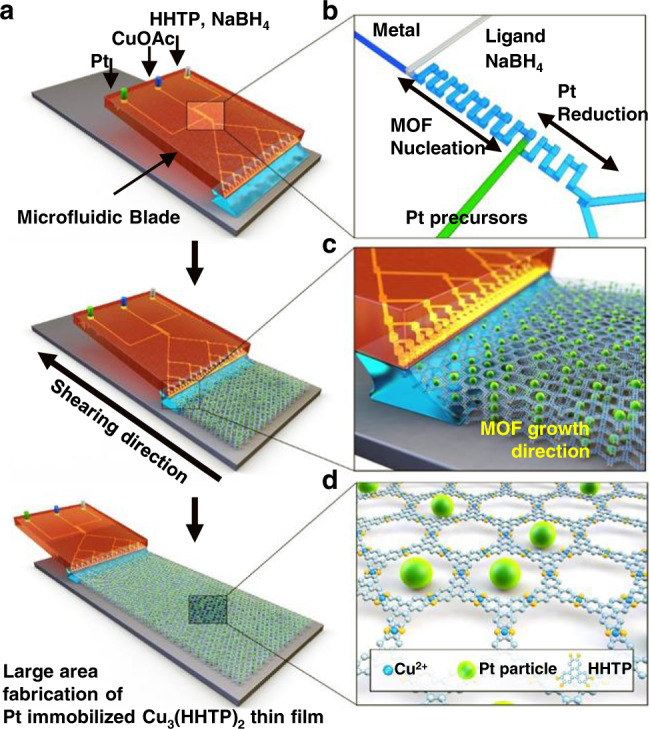
Fabrication of Pt@Cu_3_(HHTP)_2_ thin film using MiCS. **a** Schematic of Pt@Cu_3_(HHTP)_2_ MOF thin-film processing using microfluidic-based solution-shearing process. CuOAC is copper(II) acetate. **b** Schematic illustration of the microfluidic blade channel. **c** Schematic illustration of the MOF growth process using solution shearing. The nucleated Pt particle-embedded MOF solution is located between the microfluidic blade and the heated substrate. The MOF growth occurs at the edge of the meniscus. With the continuously supplying solution by the microfluidic blade during solution shearing, large-area and uniform Pt@Cu_3_(HHTP)_2_ thin film can be formed. **d** Crystal structure of Pt@Cu_3_(HHTP)_2_ thin film (green sphere: Pt particle). @ means -embedded.

Figure 1a is a schematic depiction of metal NP-embedded C-MOF thin-film formation using MiCS. Contrary to conventional solution shearing, in MiCS, microfluidic channels have been embedded within the blade, and these channels act as microreactors for the chemical synthesis of nanocatalyst-embedded C-MOFs. As one of the widely used typical C-MOFs, we chose Cu_3_(hexahydroxytriphenylene)_2_ [Cu_3_(HHTP)_2_] that has 2D structures with numerous rigid pores^3^. Cu_3_(HHTP)_2_ is generally synthesized by hydrothermal or solvothermal methods which take more than a few hours, and it has high utility in chemical sensors, supercapacitors, and other electronic devices^1–3,5^. Figure 1b is the depiction of the microfluidic channel design. Cu metal precursor solution and organic ligand (2,3,6,7,10,11-hexahydroxytriphenylene)/reducing agent (NaBH_4_) solution was firstly mixed together, inducing nucleation of Cu_3_(HHTP)_2_ C-MOF particles. It is noted that NaBH_4_ plays a role in reducing Pt precursors at the next step. This solution was then mixed with Pt precursor solution, which immobilizes the Pt nanocatalyst particles within the C-MOF pores. As the solution containing the Pt-embedded Cu_3_(HHTP)_2_ (Pt@Cu_3_(HHTP)_2_) particles flow out onto the heated substrate (150 °C), particles grow into a thin film on the substrate, as depicted in Fig. 1c and d. The gap (i.e., distance between microfluidic blade and substrate), angle, and temperature were set at 100 µm, 30°, and 150 °C, which was the optimal condition to fabricate the Pt@Cu_3_(HHTP)_2_ thin-film (Supplementary Figs. 1–3). In other words, our method induces (1) the ultrafast synthesis of C-MOFs and the reduction of metal NPs in microfluidic channels, and (2) the direct growth of metal NP-loaded C-MOFs on substrates by the solution shearing. Supplementary Table 1 summarizes the pros and cons of various MOF thin-film formation techniques. For fabrication of metal NP-embedded MOF thin films, conventional methods involve the synthesis of MOFs, the pre- or post-introduction of metal NPs (or in situ synthesis of metal NPs and MOFs), and the post process for film fabrication. However, MiCS is a unique technique where the synthesis of C-MOFs, decoration of nanocatalyst, and the growth of thin film occurs concurrently all in a single step. Therefore, our method enables the ultrafast and large-area synthesis of metal NP-embedded C-MOF thin films. In order for MiCS to work properly, the synthesis of Pt@Cu_3_(HHTP)_2_ particles must occur on the same time scale as that of the solution shearing rate. In this regard, the use of microfluidic reactors enable fast mixing and mass transfer effects unachievable by batch systems, leading to significantly enhanced reaction rates^31–33^. Hence, despite the short reaction time, continuous synthesis of Pt@Cu_3_(HHTP)_2_ particles during solution shearing is possible. Furthermore, the fast reaction rate and precisely controlled reaction time yield uniformly sized Pt NPs and Cu_3_(HHTP)_2_ particles.

**Fig. 2:**
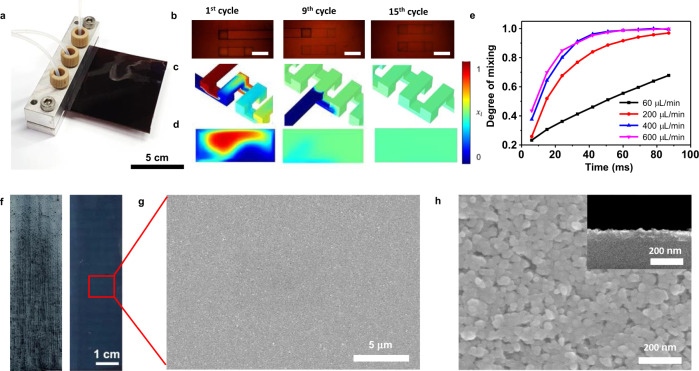
Characterization of MiCS. **a** Photographic image of the polyimide microfluidic blade. **b** Optical image of polyimide microfluidic device channel (scale bar: 500 mm): 1st cycle (left), 9th cycles (middle), 15th cycles (right). **c** 3D surface and **d** 2D cross-sectional concentration distribution of solution (${x}_{l}$) at 1st (left), 9th (middle), and 15th (right) cycle under 660 μL/min flow rate. **e** Effect of cycle number on the degree of mixing obtained at various flow rates. **f** Optical image of solution sheared Pt@Cu_3_(HHTP)_2_ film (shearing speed at 2 mm/s) on glass substrate at 150 °C: without microfluidic blade (left), and with microfluidic blade (right). **g**, SEM image of Pt@Cu_3_(HHTP)_2_ thin film. **h** High-resolution SEM image of Pt@Cu_3_(HHTP)_2_ thin film. @ means -embedded.

Figure 2a is an optical image of the microfluidic channel-embedded solution-shearing blade; further details regarding the microfluidic chip design are described in Supplementary Fig. 4. The microfluidic channels were designed as a three-dimensional serpentine mixer (Supplementary Fig. 5) due to its high mixing efficiency^34^. Figure 2b shows top view optical images of the microfluidic channels at various cycling points, and Fig. 2c and d shows the corresponding computational fluid dynamics (CFD) simulation results depicting the degree of mixing. As the number of cycles increases, the degree of mixing increases, as indicated by the appearance of the light-green-colored solution. The flow rate was optimized using CFD simulation, where various flow rates were evaluated by monitoring the degree of mixing as a function of the number of cycles (Supplementary Tables 2–5). When the flow rate exceeded 220 μL/min, 15 mixing cycles were sufficient to fully mix the solutions, as seen in Fig. 2e. At this flow rate, the solution residence time within the microfluidic channel is short (200 ms, Supplementary Fig. 6), which prevented the microfluidic channels from being clogged due to particle aggregation. Figure 2f shows optical images of the Pt@Cu_3_(HHTP)_2_ thin film using conventional solution shearing (left) and MiCS (right). In the case of solution shearing, all of the components were premixed as a bulk solution, and the solution was inserted in between the blade and the substrate prior to coating. Over a large area, the thin film coated with MiCS exhibited a high degree of uniformity without the presence of particle aggregates. This was on the contrary to thin film coated with conventional solution shearing, which visually showed variability in film color and particle aggregates. The surface roughness of the film made with MiCS was 2.8 nm, whereas that of the solution shearing was 92.3 nm (Supplementary Fig. 7). Such thin-film properties attained using MiCS can be attributed to the controlled nucleation reaction of the precursor solution within the microfluidic channel, and the continuous supply of solution that maintains constant solution volume, concentration, and the shape of the meniscus during coating. These factors can alter the fluid behavior near the meniscus, which can change the thin-film properties^35^.

### Characterizations of Pt@Cu_3_(HHTP)_2_ thin film

Figure 2g and h shows scanning electronic microscopy (SEM) images of the Pt@Cu_3_(HHTP)_2_ thin film fabricated using MiCS, confirming the closely packed MOF thin film. The average crystallite size of Pt@Cu_3_(HHTP)_2_ in the thin film is 25 nm (Supplementary Fig. 8). Powder X-ray diffraction (PXRD) was conducted on Pt@Cu_3_(HHTP)_2_ thin film grown on the substrate and on the nucleated Pt@Cu_3_(HHTP)_2_ particles being extruded out of the microfluidic channels (without undergoing thin film growth on the substrate) (Supplementary Fig. 9). Much weaker diffraction peaks were observed for the latter case, which confirms that Pt@Cu_3_(HHTP)_2_ thin-film is grown on the substrate after being extruded out of the microfluidic channels. Previous C-MOF growth techniques indicate that elevated temperature is needed for thin-film growth since energy is required to form coordination bonds; this corroborates the XRD results^36^. As stated above, the simultaneous synthesis and growth of thin film is a unique feature of MiCS, which allows the formation of a densely packed high-quality film with low surface roughness and nanoscale thickness control (discussed below).

**Fig. 3:**
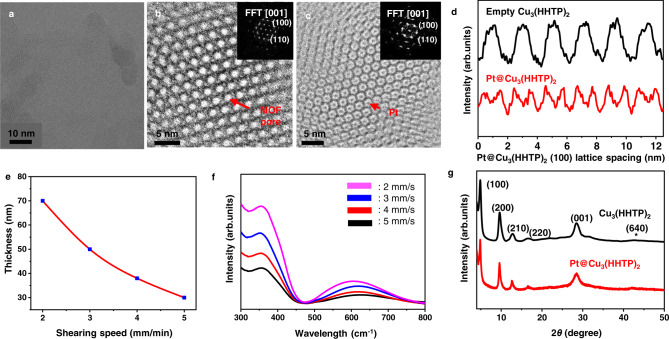
Characterization of Pt@C-MOF thin films fabricated by MiCS. **a** Cryo-TEM image of Pt@Cu_3_(HHTP)_2_ under low magnification. **b** High-resolution Cryo-TEM image of Pt@Cu_3_(HHTP)_2_ without Pt particles in the MOF pores. **c** High-resolution Cryo-TEM image of Pt particles immobilized in the MOF pores. **d** Integrated intensity of Pt@Cu_3_(HHTP)_2_ plotted over six unit cells along the [100] direction, indicating a pattern when Pt particle is introduced into the pores. **e** Thickness control of Pt@Cu_3_(HHTP)_2_ thin film via controlling the shearing speeds. **f** UV–Vis spectrum of different thicknesses of Pt@Cu_3_(HHTP)_2_ thin film. **g** PXRD data of Cu_3_(HHTP)_2_ and Pt@Cu_3_(HHTP)_2_. @ means –embedded.

To confirm the immobilization of Pt NPs within the MOF pores, cryo-transmission electronic microscopy (Cyro-TEM) was used to attain (001) plane view images of Pt@Cu_3_(HHTP)_2_, as depicted in Fig. 3a–c. Figure 3b and c is a close-up of the MOF pores in the region absent of and immobilized with Pt particles, respectively, showing a clear difference in the image contrast. Figure 3d plots the integrated pixel intensities for the empty Cu_3_(HHTP)_2_ and Pt immobilized Cu_3_(HHTP)_2_ unit cells along the [100] direction. The [100] lattice spacing (d_100_) for the empty Cu_3_(HHTP)_2_ across five unit cells was measured to be *c.a*. 2.0 nm, which is in good agreement with the theoretical pore size of Cu_3_(HHTP)_2_^22^. In the case of Pt@Cu_3_(HHTP)_2_, since the beam cannot pass through the Pt particles, darker regions within the pores were observed. To further identify the Pt particles, high-resolution-TEM (HRTEM) was used to damage the MOF structure and observe the Pt particles, as depicted in Supplementary Fig. 10. As shown in Supplementary Fig. 10a, Pt particles having an average diameter of 2 nm are well-dispersed in Cu_3_(HHTP)_2_. Supplementary Fig. 10b shows the lattice of the Pt (111) plane with a spacing of 2.265 Å. Scanning TEM (STEM) image of Pt@Cu_3_(HHTP)_2_ demonstrated that the Pt particles are well-dispersed throughout the Cu_3_(HHTP)_2_ structure (Supplementary Fig. 10c). TEM-EDS element mapping was used to confirm the presence of Cu, C, O, and Pt in the sample (Supplementary Fig. 10d). Comparing the HRTEM images of Pt@Cu_3_(HHTP)_2_ made using conventional bulk synthesis technique^15^ to that of MiCS (Supplementary Fig. 11) showed that the size variance of the Pt particles was much higher for the former. This can be attributed to the rapid and controlled Pt reduction reaction within the microfluidic channels^37^.

The thickness of Pt@Cu_3_(HHTP)_2_ thin film can be precisely controlled at tens of nanometer level using MiCS (Fig. 3e). The film thickness at various shearing speeds was confirmed using atomic force microscopy (AFM) images (Supplementary Fig. 12). The thickness decreasing with increasing shearing speed indicates that the system is in an evaporative regime, where the solvent evaporation rate is on a similar time scale to the shearing rate^38^. In this regime, thickness variation can theoretically be explained by the mass balance between the evaporating solvent and the depositing thin film to the solution flowing towards the meniscus^38^, with a power law, thickness $\propto$ speed^–1^. Our data indicate a power dependence of –0.99, which is in good agreement with the theoretical value. Ultraviolet–visible (UV/Vis) spectra were taken on Pt@Cu_3_(HHTP)_2_ films of various thicknesses, as seen in Fig. 3f. The characteristic absorbance at 363 nm (π–π* transition) and 645 nm (ligand to metal charge transfer band) increased with decreasing the shearing speed due to the increasing film thickness^14,22^. The crystal structures of Cu_3_(HHTP)_2_ and Pt@Cu_3_(HHTP)_2_ thin films were investigated by powder X-ray diffraction (PXRD) analysis (Fig. 3g). The pristine Cu_3_(HHTP)_2_ showed the crystal planes of (100), (200), (210), (220), and (001), which was similar to the observation in previous literature^10,25^. Pt@Cu_3_(HHTP)_2_ showed the same diffraction pattern as pristine Cu_3_(HHTP)_2_ without a clear appearance of Pt diffraction peak (at 2*θ* = 42^o^). It is noted that the XRD result of bulk-grown Pt@Cu_3_(HHTP)_2_ powders also displayed similar patterns with those of Pt@Cu_3_(HHTP)_2_ thin films (Supplementary Fig. 13), revealing that NaBH_4_ likely does not affect the crystal structure of C-MOF. This can be attributed to the very small amount of Pt particles and their embedment into the MOF pores^19^. The N_2_ adsorption and desorption isotherms at 77 K confirmed the porous nature of Cu_3_(HHTP)_2_ and Pt@Cu_3_(HHTP)_2_ (Supplementary Fig. 14). The Brunauer–Emmett–Teller (BET) surface area of Pt@Cu_3_(HHTP)_2_ was 200 m^2^ g^–1^, which showed a decreased value than the surface area of Cu_3_(HHTP)_2_ (250 m^2^ g^–1^). This can be ascribed to the Pt particles blocking the MOF pores^11^.

### Sensing characteristics of Pt@Cu_3_(HHTP)_2_ thin film

To demonstrate the ultrahigh catalytic activity and potential applications of Pt@Cu_3_(HHTP)_2_ thin film, we investigated its chemiresistive sensing properties. Chemiresistors are in high demand for a wide range of applications, such as environmental monitoring^39^, exhaled breath analysis^40^, and food quality control^41^. However, there are grand challenges in chemiresistive sensors: low responses to sub-ppm levels of analytes, poor cross-selectivity, and high-power consumption. We postulated that our thin films can address these issues because they have high surface area and porosity, ultrahigh reactivity, and can be operated at room temperature. In addition, since MiCS-based thin film has uniform electrical characteristics (see Supplementary Fig. 15 for comparison between MiCS-based and conventional solution-shearing-based thin-film electrical properties), relatively reliable sensing data can be attained. We fabricated chemiresistors using Pt@Cu_3_(HHTP)_2_ thin film along with control samples (Cu_3_(HHTP)_2_ powder and pristine Cu_3_(HHTP)_2_ thin film) (see details in the “Methods”). First, to optimize the NO_2_ sensing properties of Pt@Cu_3_(HHTP)_2_ thin-film-based sensors at room temperature in air, we controlled the loading amounts of Pt NPs by the different flow rates of Pt precursors (50, 100, 150, and 200 μL/min) (Supplementary Fig. 16). The weight ratio of Pt-loaded Cu_3_(HHTP)_2_ was calculated using Inductively Coupled Plasma Mass Spectrometer analysis (ICP-MS) (Supplementary Table 6). Among the samples, the 2.3 wt% Pt-loaded Cu_3_(HHTP)_2_ thin film exhibited the optimum NO_2_ sensing properties (hereafter, the 2.3 wt% Pt-loaded Cu_3_(HHTP)_2_ thin film represents the Pt@Cu_3_(HHTP)_2_ thin-film-based NO_2_ sensors).

**Fig. 4:**
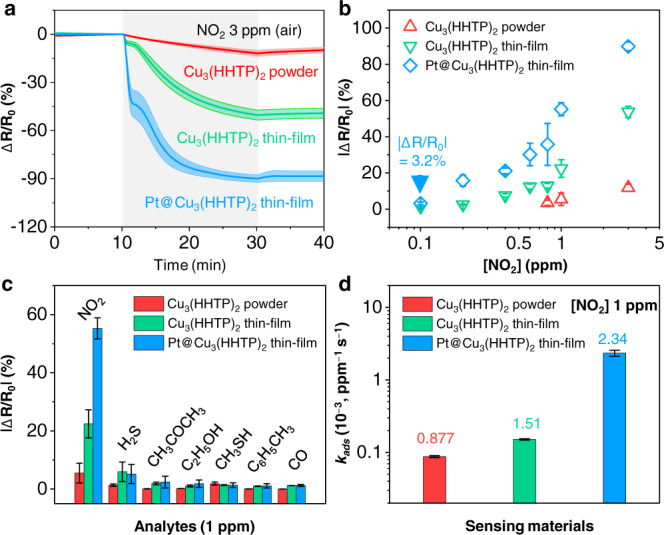
Sensing characteristics of Pt@Cu_3_(HHTP)_2_ thin film (shearing speed: 2 mm/s) on alumina (Al_2_O_3_) substrate at 150 °C. **a** Resistance changes of Cu_3_(HHTP)_2_ powder, Cu_3_(HHTP)_2_ thin film, and Pt@Cu_3_(HHTP)_2_ thin film in response to 20 min exposure of NO_2_ 3 ppm. The shaded areas present the standard deviation of the sensors (*N* ≥ 4). **b** Normalized responses of the sensors to 0.1–3 ppm of NO_2_. **c** Selectivity of the sensors to 1 ppm of analytes. **d** Calculated adsorption rate constants (*k*_ads_) of the sensors. @ means –embedded. The error bars in **b–d** show the standard deviation of the sensors (*N* ≥ 4).

The Pt@Cu_3_(HHTP)_2_ thin film displays significant resistance changes (ΔR/R_a_ = –89.9%) at 3 ppm of NO_2_ (Fig. 4a). In contrast, the Cu_3_(HHTP)_2_ powder and Cu_3_(HHTP)_2_ thin film show lower responses (–11.8% for Cu_3_(HHTP)_2_ powder and –53.7% for Cu_3_(HHTP)_2_ thin film) relative to the Pt@Cu_3_(HHTP)_2_ thin film. In addition, the Pt@Cu_3_(HHTP)_2_ and Cu_3_(HHTP)_2_ thin films are able to detect NO_2_ as low as 0.1 ppm, whereas the Cu_3_(HHTP)_2_ powder do not function below 1 ppm (Supplementary Fig. 17). We calculated normalized responses (|ΔR/R_0_ | ) and response times (*t*_90_) of the sensors in the range of NO_2_ 0.1–3 ppm (Fig. 4b and Supplementary Fig. 18). The formation of thin-film structure (Cu_3_(HHTP)_2_ thin film) improves the responses of Cu_3_(HHTP)_2_ by a factor of 4, compared to Cu_3_(HHTP)_2_ powder. In addition to the structural effect, the decoration of Pt NPs (Pt@Cu_3_(HHTP)_2_ thin film) induces twofold higher responses than pristine Cu_3_(HHTP)_2_ thin-film. Furthermore, the Pt@Cu_3_(HHTP)_2_ thin-film shows faster-sensing speed (8.2 min to NO_2_ 3 ppm) compared to the Cu_3_(HHTP)_2_ thin-film (14 min) and Cu_3_(HHTP)_2_ powder (17.3 min). These results demonstrated that both the thin-film structure of Cu_3_(HHTP)_2_ and ultra-small Pt NPs increase the activity of the sensors. Although the response times of the Pt@Cu_3_(HHTP)_2_ thin film are sluggish at low NO_2_ concentrations (<1 ppm), the Pt@Cu_3_(HHTP)_2_ thin-film-based sensors (13 min for NO_2_ 1 ppm) meet the safety standard for NO_2_ exposures; the short-term permissible exposure limit of NO_2_, designated by the Occupational Safety and Health Administration (OSHA) in the USA, is 1 ppm within 15 min.

We further investigated the other important parameters for NO_2_ sensors: selectivity and stability. The Pt@Cu_3_(HHTP)_2_ thin-film sensors display ultrahigh NO_2_ cross-selectivity against other interfering analytes (hydrogen sulfide [H_2_S], acetone [CH_3_COCH_3_], ethanol [C_2_H_5_OH], methanethiol [CH_3_SH], toluene [C_6_H_5_CH_3_], and carbon monoxide [CO]) (Fig. 4c). The responses (55.3%) of the Pt@Cu_3_(HHTP)_2_ thin film to NO_2_ are tenfold higher than those (>5.2%) to other gas molecules. In addition, the sensors show stable responses to multiple exposures to NO_2_ 0.1 ppm (Supplementary Fig. 19a), demonstrating high feasibility as dosimetric sensors. Furthermore, the Pt@Cu_3_(HHTP)_2_ thin-film-based sensors function well in humid air (relative humidity = 95%) (Supplementary Fig. 19b). There are slight decreases in the response (–38.9% to NO_2_ 1 ppm) and response speed (18 min) under the humid air (relative humidity: 90%) compared to those (response = –55.3% and response time = 13 min) under the dry air (relative humidity: 5%). These decreases are attributed to the fact that adsorbed water molecules retard the reactions of NO_2_ on Pt@Cu_3_HHTP_2_ thin film. Nonetheless, these sensing performances demonstrate that our sensors represent a viable NO_2_ sensor operated at room temperature in air.

**Fig. 5:**
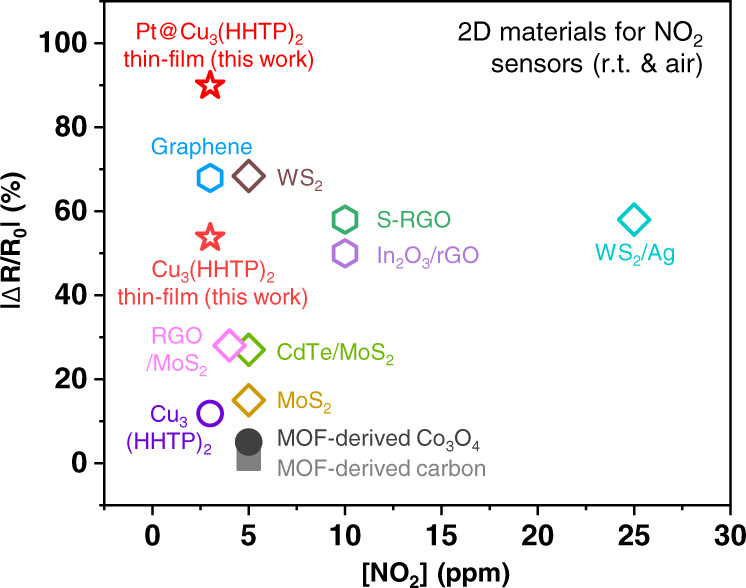
Comparison of the NO_2_ responses of Pt@Cu_3_(HHTP)_2_ thin-film and Cu_3_(HHTP)_2_ thin-film with those of other materials. Other materials include Cu_3_(HHTP)_2_, graphene, WS_2_, RGO/MoS_2_ (RGO: reduced graphene oxide), CdTe/MoS_2_, MoS_2_, MOF-derived CO_3_O_4_, MOF-derived carbon, S-RGO (S-RGO: sulfonated RGO), In_2_O_3_/RGO, and WS_2_/Ag. / means heterostructured with.

To elucidate the structural and catalytic effect of Pt@Cu_3_(HHTP)_2_ thin film on NO_2_ sensing, we calculated adsorption rate kinetics (*k*_ads_) using response traces of the sensors. Since the origin of chemiresistive sensing of Cu_3_(HHTP)_2_ is charge transfers caused by adsorbed gas molecules^12,42^, with the assumption that the responses of Cu_3_(HHTP)_2_ are proportional to the amounts of adsorbed NO_2_ molecules, reaction rate constants are obtained by fitting response traces to theoretical equations (see details in Supplementary Fig. 20 and Supplementary Table 7). The calculated *k*_ads_ values are described in Fig. 4d. The Pt@Cu_3_(HHTP)_2_ thin film shows higher NO_2_ adsorption kinetics (*k*_ads_ = 2.34 × 10^−3^ ppm^−1^ s^−1^) than the Cu_3_(HHTP)_2_ powder (0.877 × 10^−3^ ppm^−1^ s^−1^) and the Cu_3_(HHTP)_2_ thin film (1.51 × 10^−3^ ppm^−1^ s^−1^), demonstrating that NO_2_ reactions on Cu_3_(HHTP)_2_ are promoted by two factors: (1) the thin-film structure of Cu_3_(HHTP)_2_ and (2) the catalytic effect of ultra-small Pt NPs. The thin-film structure induces high gas accessibility into sensing layers^14^, and ultra-small Pt NPs (~2 nm) cause NO_2_ spillover onto Cu_3_(HHTP)_2_^15^. Therefore, NO_2_ molecules are easily accessible to Cu_3_(HHTP)_2_ layers, and their reactions are activated by nanoscopic Pt catalysts, leading to high sensing performances. These sensing performances are higher than other 2D material-based NO_2_ chemiresistors operated at room temperature in air (Fig. 5 and Supplementary Table 8)^15,43–52^. In particular, the NO_2_ responses of our sensors are hugely improved compared to MOF-based NO_2_ sensors.

## Discussion

C-MOFs are emerging materials with a high degree of chemical versatility and ultrahigh porosity, rendering them a suitable material for next-generation electronics, energy storage devices, and sensors. To fully take advantage of C-MOFs, the generation of high-quality nanoscale thin films with the versatility to immobilize nanocatalysts into the MOF pores is of critical importance. This has thus far been a major challenge as the simultaneous synthesis of MOF thin-film and immobilization of nanocatalysts is difficult. Furthermore, high-throughput, large-area, single-step (synthesis, film growth, catalyst immobilization) thin-film generation remains challenging, limiting the commercial feasibility of C-MOFs. Our proposed MiCS technique overcomes these difficult challenges. The use of microfluidic channels enables the synthesis of catalyst-embedded MOF particles, which simultaneously grow into high-quality nanoscale thin-film in a large-area scalable, high-throughput manner. Our thin films exhibit significantly improved gas sensing performance, as a consequence of (1) the high porosity and facile gas accessibility of C-MOF thin films, and (2) the ultrahigh catalytic activity of the embedded NPs (c.a. 2 nm). These results together point to the exciting prospects of utilizing MiCS for the generation and optimization of a variety of catalyst-embedded C-MOF thin films for various applications in the future.

## Methods

### Materials

All chemicals and solvents were of reagent grade and were used as received without further purification. Copper(II) acetate monohydrate (Cu(CO_2_CH_3_)H_2_O, 99.9%), dimethyl sulfoxide (DMSO), potassium tetrachloroplatinate(II) (K_2_PtCl_4_), and sodium borohydride (NaBH_4_, 96%) were purchased from Sigma-Aldrich. 2,3,6,7,10,11-hexahydroxytriphenylene hydrate (HHTP, C_18_H_12_O_6_H_2_O, 95.0%) was purchased from Tokyo Chemical Industry. Glass, silicon wafer, and alumina (Al_2_O_3_) were used as a substrate, and all substrates were treated with oxygen plasma for 1 min prior to coating of MOF thin-film.

### Mathematical formulation and numerical details

The flow behavior and mixing progress in the microfluidic mixing region were investigated numerically based on the finite element method (FEM) with COMSOL Multiphysics software (v5.5, Comsol Inc.). The computation of steady-state fluid flow was followed by the mass transfer analysis based on the derived velocity profile.

The flow regime (${Re}{\mathscr{ \sim }}{\mathscr{O}}\left(10\right)\ll 2000$) for this work was considered as laminar flow, so the turbulent model was not involved^34^. The velocity (${\bf{v}}$) and pressure ($p$) profile were derived by numerically solving the governing equations as below:

1 $$\rho ({\bf{v}}\cdot {\boldsymbol{\nabla }}){\bf{v}}=-{\boldsymbol{\nabla }}\cdot [-p{\bf{I}}+{\bf{K}}]{\rm{:Navier}}-{\rm{Stokes}}\; {\rm{equation}},$$

2 $${\boldsymbol{\nabla }}\cdot {\bf{v}}=0{{:}}{\rm{continuity}}\;{\rm{equation}},$$

3 $${\bf{K}}=\mu \left({\boldsymbol{\nabla }}{\bf{v}}+{\left({\boldsymbol{\nabla }}{\bf{v}}\right)}^{{\rm{T}}}\right){{:{\rm{constitutive}}\;{\rm{equation}}}},$$

where the density ($\rho$) and viscosity ($\mu$) followed the solvent’s properties, dimethyl sulfoxide (DMSO, whose $\rho =1.1{\rm{g}}/{\rm{mL}}$ and $\mu =2{\rm{mPa}}\cdot {\rm{s}}$). Concentration dependence on viscosity and density could be neglected based on the comparison of calculation results under similar conditions in the previous work^34^. As boundary conditions, the mass flow rate for inlet, nonslip condition on the inner surface of the wall, and ambient air pressure on the outlet were applied.

The concentration change of NaBH_4_ ligand (${C}_{{\rm{l}}}$), CuOAc metal (${C}_{{\rm{m}}}$), and Pt precursors (${C}_{{\rm{Pt}}}$) in DMSO were derived by solving governing equations for mass transfer as below:

4 $${\boldsymbol{\nabla }}\cdot {{\bf{J}}}_{{\rm{i}}}+{\bf{v}}\cdot {\boldsymbol{\nabla }}{c}_{{\rm{i}}}={R}_{{\rm{i}}}{\rm{:mass}}\; {\rm{conservation}}\; {\rm{equation}},$$

5 $${{\bf{J}}}_{{\rm{i}}}=-D{\boldsymbol{\nabla }}{c}_{{\rm{i}}}{\rm{:Fick}}'{\rm{s}}\; {\rm{diffusion}}\; {\rm{equation}},$$

where the generic molecular diffusivity (10^−9^ m^2^/s) were applied for the molecular diffusivity ($D$). Because the Péclet number for this study is in the range of ${10}^{3}\le {Pe}\le 2\times {10}^{4}$, convection-mediated mass transfer dominates mass transfer. Nucleation and catalyst embedding are sufficiently fast to assume that the degree of mixing directly indicates the conversion of HTTP nucleation and Pt reduction. In other words, under steady-state conditions, the right-hand side of the mass conservation equation was considered zero. For the boundary condition of mass transfer, the individual initial concentrations for each inlet and no mass flux gradient for the outlet were applied.

The geometry exactly the same as the microfluidic blade was manually constructed and was divided into computational elements in COMSOL, where the number of elements was determined by the mesh size dependency test. To achieve adequate calculation accuracy within the limits allowed by computational power (2.1 GHz 24 core CPU, 250 GB RAM), first, second, and quadratic order discretization was applied for pressure, velocity, and concentration, individually.

### Microfluidic cycle and degree of mixing (${\boldsymbol{\varepsilon }}$)

For indication and evaluation, the entire microfluidic blade was divided into microfluidic cycles as defined in Supplementary Fig. 5. Except for the first cycle, each cycle includes four turns and experiences the same residence time determined by the mass flow rate. The plane between the *n*th cycle and *n* + 1th cycle is defined as P_*n*+1_ (*n* $\ge$ 1), and P_1_ is considered as the plane where the ligand and metal first meet, so the first cycle has only two turns after P_1_.

The degree mixing was calculated at each plane (P_*n*_) showing the result of each *n*th cycle. From the numerical solution of each solutes’ concentration, the molar fraction of ligand (${x}_{{\rm{l}}}=\frac{{c}_{{\rm{l}}}}{\mathop{\sum}\limits_{{\rm{i}}}{c}_{{\rm{i}}}},{\rm{i}}={\rm{l}},{\rm{m}},\,{\rm{and}}\; {\rm{Pt}}$) is used in order to quantify the degree of mixing which directly denotes the progress of MOF nucleation and Pt reduction. The degree of mixing ($\varepsilon$) is defined with its standard deviation of ${x}_{{\rm{l}}}$ ($\sigma$), as below:

6 $$\varepsilon =1-\frac{\sigma }{{\sigma }_{{{\max }}}}{\rm{:degree}}\; {\rm{of}}\; {\rm{mixing}}.$$

The maximum standard deviation (${\sigma }_{{{\max }}}$) was designated at the plane where the ligand or Pt precursor and metal meet first (i.e., ${\sigma }_{{{\max }}}=0.498$ for ligand-metal and 0.287 for ligand–Pt precursor at P_1_).

### Fabrication of microfluidic blade

First, PI films of 125 μm in thickness (Kapton HN film, Dupont, USA) were ablated using UV laser (355 nm, ESI, USA) to form the desired micropatterns (300 μm in width). In laser, ablated regions were completely eliminated from the PI films. Thereafter, FEP (fluoroethylene propylene) nanopowder dispersed in an aqueous solution was spin-coated (2000 rpm, 50 s) onto each of the PI films. Then the FEP-coated PI films were vertically stacked using a metal holder with aligners. Finally, the aligned PI films were mechanically pressed at 350 °C under a pressure of 10 kPa for 3 h.

### Synthesis of Cu_3_(HHTP)_2_ film using MiCS process

A solution of Copper(II) acetate monohydrate (0.12 M in DMSO, 200 μL/min) and a solution of HHTP (0.1 M in DMSO, 200 μL/min) were introduced into two inlets of the microfluidic blade continuously using syringe pumps (Harvard Apparatus PHD 4400) at a rate of 400 μL/min. The microfluidic channels were shaped as a three-dimensional serpentine structure with a total of 15 cycles of mixing to induce effective mixing. The reacted solution is discharged through the 64-outlets of the microfluidic blade onto the heated substrate (150 °C). The gap (i.e., the distance between microfluidic blade and substrate) and angle were set at 100 μm and 30°, respectively. The generated Cu_3_(HHTP)_2_ thin-film was washed with DMSO and ethanol, respectively.

#### Synthesis of Pt@Cu_3_(HHTP)_2_ film using MiCS process

A solution of copper(II) acetate monohydrate (0.12 M in DMSO, 200 μL/min) and a mixed solution of HHTP with NaBH_4_ (HHTP: 0.1 M in DMSO, NaBH_4_: 1 mg mL^−1^, 200 μL/min) were introduced to two inlets of the microfluidic blade using syringe pumps. Pt solution (1 mg mL^−1^, 50, 100, 150, and 200 μL/min) was inserted at the 9th cycle of mixing. The resulting solution is discharged continuously between the microfluidic blade and the heated substrate (150 °C). The gap (i.e., the distance between the microfluidic blade and substrate) and angle were set at 100 μm and 30°_,_ respectively. The generated Pt@Cu_3_(HHTP)_2_ thin-film was washed with DMSO and ethanol, respectively.

#### Material characterization

Cryo Field Emission TEM (Glacios, Thermo Fisher) at 200 kV, field-emission TEM (Tecnai G2 S-Twin, FEI) at 300 kV, and spherical aberration-corrected TEM (JEMARM200F, JEOL) at 200 kV were conducted to investigate the microstructure of the samples. The MOF thin films were imaged using a field-emission scanning-electron microscope (FE-SEM, Hitachi S-4800). X-ray diffraction (XRD) patterns were measured by D/Max-2500 (RIGAKU) diffractometer. Film thickness and topology were measured with tapping mode AFM (AFM WORKSHOP, PS-2010). The nitrogen adsorption-desorption isotherms were obtained using a BELSORP-max at 77 K. Prior to the adsorption measurements, all samples were collected by a thick layer of MOF films (~20 mg) and were evacuated (*P* < 10^–5^ mbar) at 393 K for 5 h. To obtain 20 mg of the samples using our method, we fabricated 15–20 thick films (shearing size: about 45 × 60 mm). The specific surface area was obtained by the Brunauer–Emmett–Teller (BET) method.

### Sensing measurements

Cu_3_(HHTP)_2_ and Pt@Cu_3_(HHTP)_2_ thin films were directly formed on alumina (Al_2_O_3_) substrate (2.5 mm (width) × 2.5 mm (length) × 0.2 mm (thickness)). Bulk Cu_3_(HHTP)_2_-based sensors were prepared by drop-coating suspension of the Cu_3_(HHTP)_2_ powder (5 mg in 300 µL of ethanol) onto the alumina substrate. To trace the resistance of the sensors, Au electrodes with a gap of 70 μm were deposited on top of the films. NO_2_ sensing tests were carried out at room temperature in air. Before the sensing tests, the sensors were stabilized by fresh air for 4 h. Thereafter, the sensors were exposed to NO_2_ for 20 min, with the NO_2_ concentrations controlled in the range of 0.1–3 ppm using a mass flow controller. Resistance of the sensors was monitored using an acquisition system (34972, Agilent) in real time. The response was defined as a ratio of the resistance change (Δ*R*) to the resistance in the air (*R*_0_). Response time (*t*_90_) was the set as the time taken to reach 90% of the maximum resistance change (0.9Δ*R*_max_).

## Supplementary information

Supplementary information

## Data Availability

The authors declare that the data supporting the findings of this study are available within the article and its Supplementary Information files. Extra data are available from the corresponding author upon reasonable request.
