# Supplementary material for: Large-area synthesis of nanoscopic catalyst-decorated conductive MOF film using microfluidic-based solution shearing
Source: Nat Commun. 2021 Jul 13;12:4294. doi: 10.1038/s41467-021-24571-1 (PMC8277906; doi:10.1038/s41467-021-24571-1)
Supplement: Supplementary file 1 — Supplementary information [file 41467_2021_24571_MOESM1_ESM.pdf]

## Supplementary Information

# Large-area synthesis of nanoscopic catalyst-decorated conductive MOF film using microfluidic-based solution shearing

Jin-Oh Kim[1,5],Won-Tae Koo[1,2,5], Hanul Kim[3], Chungseong Park[1,2], Taehoon Lee[1], Calvin Andreas Hutomo[1], Siyoung Q. Choi[3], Dong Soo Kim[4], Il-Doo Kim[1,2,*] and Steve Park[1,*]

[1] Department of Materials Science and Engineering, Korea Advanced Institute of Science and Technology (KAIST), 291 Daehak-ro, Yuseong-gu, Daejeon 34141, Republic of Korea

[2] Membrane Innovation Center for Anti-virus & Air-quality Control, KAIST Institute for Nanocentury, 291, Daehak-ro, Yuseong-gu, Daejeon 34141, Republic of Korea

[3] Department of Chemical and Biomolecular Engineering, Korea Advanced Institute of Science and Technology (KAIST), Daejeon 34141, Korea

[4] Department of Creative Convergence Engineering, Hanbat National University, Daejeon 34158, Korea

[5] These authors contributed equally: Jin-Oh Kim, Won-Tae Koo.
*email: idkim@kaist.ac.kr; stevepark@kaist.ac.kr

**Table of Contents**

**- Supplementary Table 1.** Summary of various fabrication methods of ultrathin C–MOFs.

**- Supplementary Table 2.** Mixing index and the concentration distribution of ligand for 10 microfluidic cycles without Pt solution's flow.

**- Supplementary Table 3.** 3D surface and 2D cross sectional concentration distribution of ligand for 10 microfluidic cycles without Pt solution's flow.

**- Supplementary Table 4.** Mixing index and the concentration distribution of ligand for 15 microfluidic cycles with Pt solution's flow.

**- Supplementary Table 5.** 3D surface and 2D cross sectional concentration distribution of ligand for 15 microfluidic cycles with Pt solution's flow.

**- Supplementary Table 6.** The weight ratio of Cu and Pt in Pt@$Cu_3(HHTP)_2$ at various flow rates of Pt precursors.

**- Supplementary Table 7.** Summary of the reaction rate constants of the sensors.

**- Supplementary Table 8.** Summary of sensing properties of 2D material-based $NO_2$ sensors.

**- Supplementary Fig. 1.** Optimal condition of Pt@$Cu_3(HHTP)_2$ film at various distances between microfluidic blade and substrate.

**- Supplementary Fig. 2.** Optimal condition of Pt@$Cu_3(HHTP)_2$ film at various angle between microfluidic blade and substrate.

**- Supplementary Fig. 3.** Optimal condition of Pt@$Cu_3(HHTP)_2$ film at various temperature.

**- Supplementary Fig. 4.** Fabrication of microfluidic blade.

**- Supplementary Fig. 5.** Computational fluid dynamics simulation of microfluidic blade

**- Supplementary Fig. 6.** Effect of mixing time on the degree of mixing at various flow rates.

**- Supplementary Fig. 7.** Surface roughness profile measurement of Pt@$Cu_3(HHTP)_2$ thin-film.

**- Supplementary Fig. 8.** Size distribution of Pt@$Cu_3(HHTP)_2$ crystallite.

**- Supplementary Fig. 9.** XRD patterns of Pt@$Cu_3(HHTP)_2$ after MiCS process, and only after microfluidic synthesis process without solution shearing.

**- Supplementary Fig. 10.** TEM images of Pt@$Cu_3(HHTP)_2$

**- Supplementary Fig. 11.** HRTEM image of Pt@$Cu_3(HHTP)_2$ under different synthesis processes.

**- Supplementary Fig. 12.** AFM topographic images of a manually scratched Pt@$Cu_3(HHTP)_2$ films at various shearing speeds.

**- Supplementary Fig. 13.** XRD analysis of $Cu_3(HHTP)_2$ powders and Pt@$Cu_3(HHTP)_2$ powders.

**- Supplementary Fig. 14.** $N_2$ adsorption and desorption isotherms of the $Cu_3(HHTP)_2$ and Pt@$Cu_3(HHTP)_2$ samples at 77 K.

**- Supplementary Fig. 15.** Comparison of electrical properties of films fabricated through MiCS process and conventional solution shearing process.

**- Supplementary Fig. 16.** Responses of Pt@$Cu_3(HHTP)_2$ thin-film at different ratios of Pt NPs.

- **Supplementary Fig. 17.** Response traces of the sensors to 0.1–1 ppm of $NO_2$.

- **Supplementary Fig. 18.** Response times of the sensors to 0.1–3 ppm of $NO_2$.

- **Supplementary Fig. 19.** Additional sensing results.

- **Supplementary Fig. 20.** Exponential fitting curves of the sensors.

- **References**

**Supplementary Table 1.** Summary of various fabrication methods of C-MOF-based films.

| Methods | Example materials | Process times | THK[a] | Conductivity (@RT[b]) | Pros | Cons | Ref[c] |
|---|---|---|---|---|---|---|---|
| Drop casting | $Cu_3(HITP)_2$ | 1. Synthesis: ~2 days<br>2. Deposition: ~30 min | ≥ μm | $2 \times 10^{-1}$ S/cm | • Facile processes<br>• Applicable to various supports | • Poor uniformity<br>• High surface roughness<br>• Difficult to produce ultra-thin layers<br>• Long processing time<br>• Difficult to synthesize at large-scale with high uniformity | 1, 2, 3 |
| | $Ni_3(HITP)_2$ | | | 2 S/cm | | | |
| | $Cu_3(HHTP)_2$ | | | $2 \times 10^{-3}$ S/cm | | | |
| | $Ni_3(HHTP)_2$ | | | $1 \times 10^{-2}$ S/cm | | | |
| Layer-by-layer (LbL) growth | $Cu_3(HHTP)_2$ | 1. Preparing substrates: ~1 days<br>2. LbL growth: ~1 min/cycle | ~2 nm/ cycle | $2 \times 10^{-2}$ S/cm | • High uniformity<br>• Accurate control of film THK<br>• Ultra-thin structure | • Need of chemical modification on substrates<br>• Sequential LbL steps that can lead to long processing time | 1, 4 |
| Liquid-liquid interfacial reaction | $Ag_3BHT_2$ | 30 min | ~280 nm | 363 S/cm | • Fast synthesis<br>• High uniformity | • Difficult to control THK of films<br>• Difficult to synthesize at large-scale with high uniformity | 5 |
| | $Au_3BHT_2$ | 6.5 h | ~320 nm | | | | |
| Microfluidic-based solution shearing (MiCS) | $Cu_3(HHTP)_2$ | 10 min | ~100 nm | $2 \times 10^{-2}$ S/cm | • Facile synthesis<br>• Ultra-fast synthesis<br>• High uniformity<br>• Accurate control of film THK<br>• Ultra-thin structure<br>• Large scale synthesis<br>• Simultaneous incorporation of nanocatalyst in the pores. | | This work |
| | $Pt@Cu_3(HHTP)_2$ | | | $1 \times 10^{-3}$ S/cm | | | |

THK[a] is the thickness of the C-MOF-based films. RT[b] is room temperature. Ref[c] is reference.

**Supplementary Table 2**. Mixing index and the concentration distribution of ligand for 10 microfluidic cycles without Pt solution's flow.

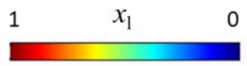


| Number of Cycles | Mixing index | | | |
|---|---|---|---|---|
| | 30 μL/min | 100 μL/min | 200 μL/min | 300 μL/min |
| 1 | 0.23 | 0.26 | 0.38 | 0.44 |
| 2 | 0.31 | 0.52 | 0.64 | 0.70 |
| 3 | 0.36 | 0.68 | 0.80 | 0.85 |
| 4 | 0.41 | 0.77 | 0.91 | 0.90 |
| 5 | 0.46 | 0.84 | 0.96 | 0.95 |
| 6 | 0.51 | 0.89 | 0.98 | 0.97 |
| 7 | 0.56 | 0.92 | 0.99 | 0.99 |
| 8 | 0.60 | 0.94 | 0.99 | 0.99 |
| 9 | 0.64 | 0.96 | 1.00 | 1.00 |
| 10 | 0.68 | 0.97 | 0.99 | 1.00 |
| Concentration distribution at $P_2$ | 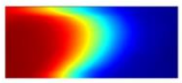 | 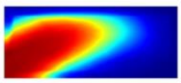 | 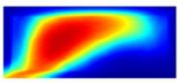 | 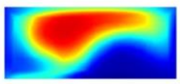 |
| Concentration distribution at $P_4$ | 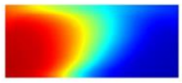 | 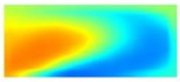 | 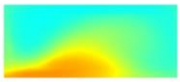 | 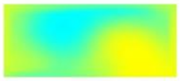 |

**Supplementary Table 3**. 3D surface and 2D cross sectional concentration distribution of ligand for 10 microfluidic cycles without Pt solution's flow.

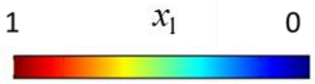


| Number of cycles | Concentration of ligand | | | | | | | |
|---|---|---|---|---|---|---|---|---|
| | 30 µL/min | | 100 µL/min | | 200 µL/min | | 300 µL/min | |
| | 3D surface | Cross section | 3D surface | Cross section | 3D surface | Cross section | 3D surface | Cross section |
| $1^{st}$-$2^{nd}$ cycle | 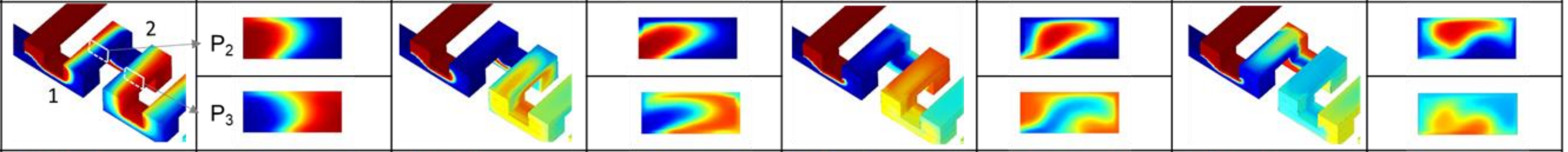  | | | | | | | |
| $3^{rd}$-$4^{th}$ cycle | 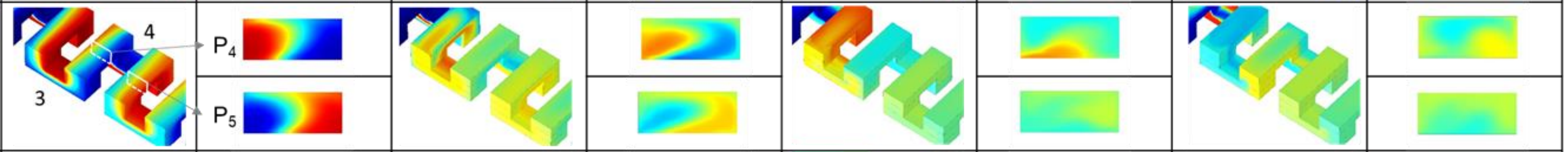  | | | | | | | |
| $5^{th}$-$6^{th}$ cycle | 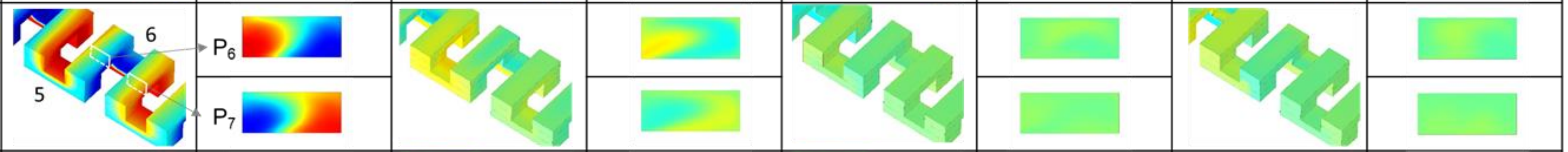  | | | | | | | |
| $7^{th}$-$8^{th}$ cycle | 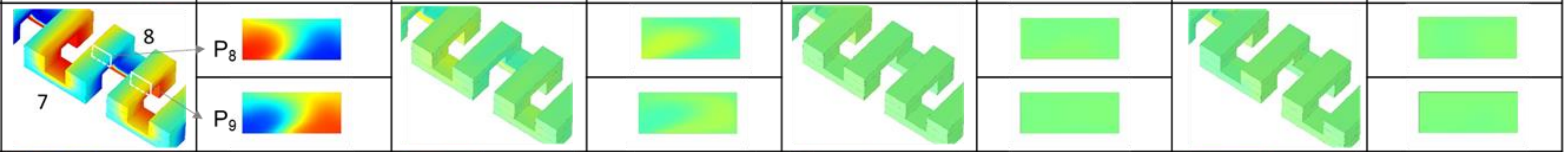  | | | | | | | |
| $9^{th}$-$10^{th}$ cycle | 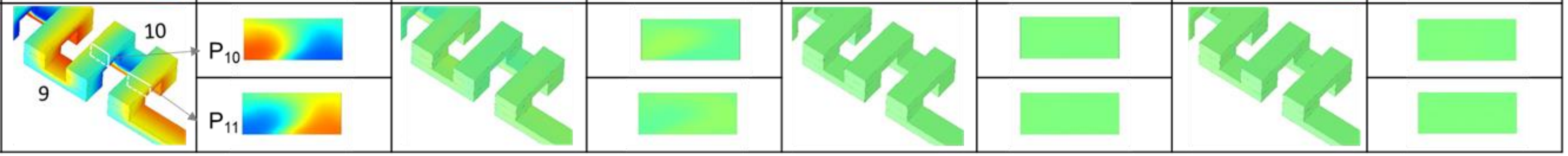  | | | | | | | |

**Supplementary Table 4**. Mixing index and the concentration distribution of ligand for 15 microfluidic cycles with Pt solution's flow.

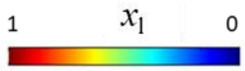


| Number of cycles | Mixing index | | | |
|---|---|---|---|---|
| | 30 μL/min | 100 μL/min | 200 μL/min | 300 μL/min |
| 1 | 0.25 | 0.30 | 0.40 | 0.47 |
| 2 | 0.33 | 0.56 | 0.68 | 0.75 |
| 3 | 0.39 | 0.72 | 0.84 | 0.89 |
| 4 | 0.45 | 0.82 | 0.94 | 0.94 |
| 5 | 0.51 | 0.88 | 0.97 | 0.97 |
| 6 | 0.56 | 0.92 | 0.99 | 0.99 |
| 7 | 0.60 | 0.95 | 0.99 | 0.99 |
| 8 | 0.65 | 0.97 | 1.00 | 1.00 |
| 9 | 0.70 | 0.98 | 1.00 | 1.00 |
| 10 | 0.73 | 0.98 | 1.00 | 1.00 |
| 11 | 0.77 | 0.98 | 0.99 | 1.00 |
| 12 | 0.81 | 0.98 | 0.99 | 0.99 |
| 13 | 0.84 | 0.99 | 1.00 | 1.00 |
| 14 | 0.86 | 1.00 | 1.00 | 1.00 |
| 15 | 0.88 | 1.00 | 1.00 | 1.00 |
| Concentration distribution at $P_2$ | 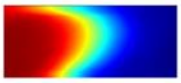 | 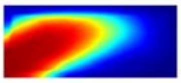 | 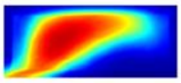 | 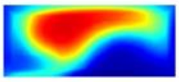 |
| Concentration distribution at $P_4$ | 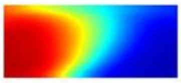 | 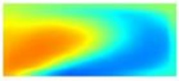 | 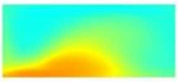 | 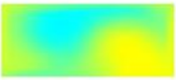 |
| Concentration distribution at $P_{12}$ | 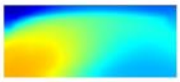 | 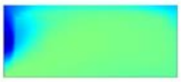 | 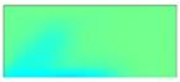 | 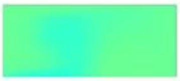 |

**Supplementary Table 5**. 3D surface and 2D cross sectional concentration distribution of ligand for 15 microfluidic cycles with Pt solution's flow.

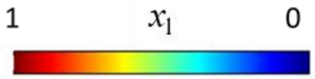


| Number of cycles | Concentration of ligand | | | | | | | |
|---|---|---|---|---|---|---|---|---|
| | 30 µL/min | | 100 µL/min | | 200 µL/min | | 300 µL/min | |
| | 3D surface | Cross section | 3D surface | Cross section | 3D surface | Cross section | 3D surface | Cross section |
| $1^{st}$-$2^{nd}$ cycle | 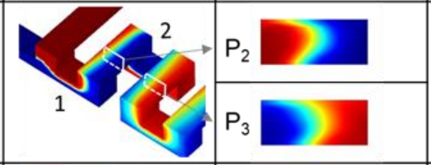  | | 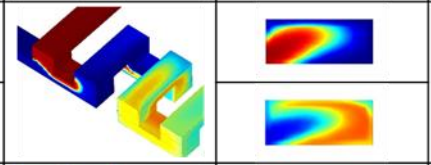 | | 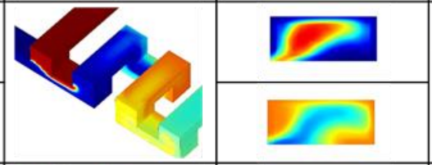 | | 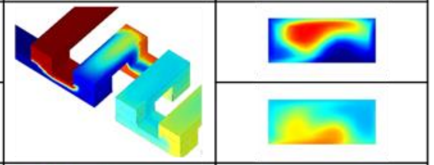 | |
| $8^{th}$-$9^{th}$ cycle | 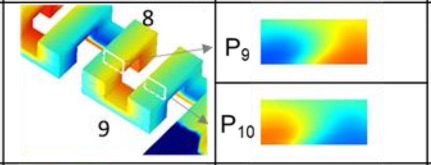  | | 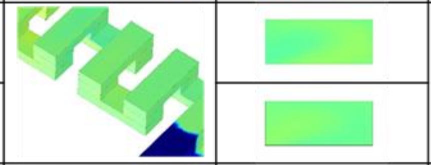 | | 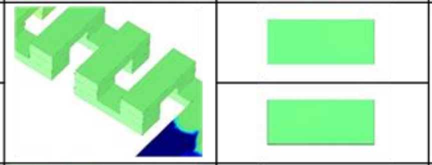 | | 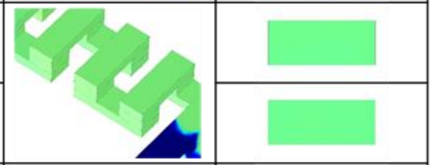 | |
| $10^{th}$-$11^{th}$ cycle | 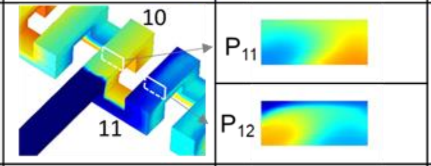  | | 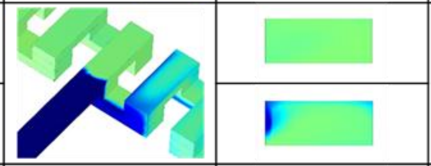 | | 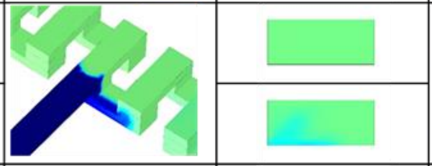 | | 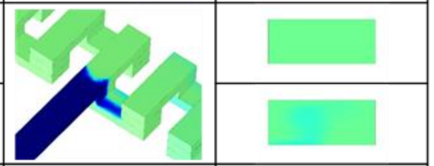 | |
| $12^{th}$-$13^{th}$ cycle | 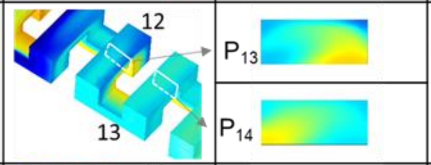  | | 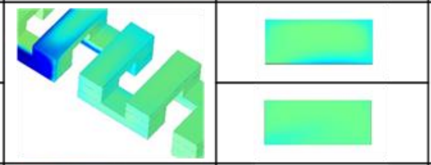 | | 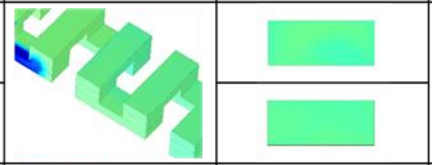 | | 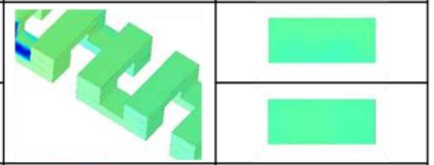 | |
| $14^{th}$-$15^{th}$ cycle | 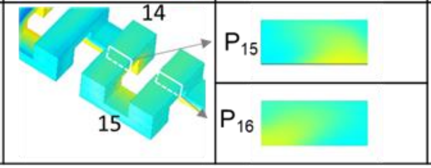  | | 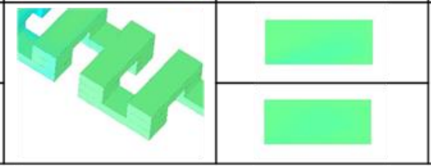 | | 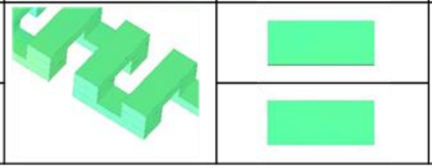 | | 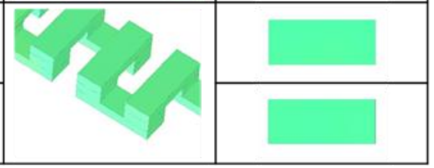 | |

**Supplementary Table 6.** The weight ratio of Cu and Pt in Pt@$Cu_3(HHTP)_2$ at various flow rates of Pt precursors.

| Flow rate (μL/min) | Cu (Weight ratio %) | Pt (Weight ratio %) |
|---|---|---|
| 50 | 20.1 | 0.9 |
| 100 | 20.0 | 2.3 |
| 150 | 20.2 | 2.7 |
| 200 | 20.4 | 3.5 |

**Supplementary Table 7.** Summary of the reaction rate constants of the sensors.

| | $k_{ads}$ (ppm$^{-1}$ s$^{-1}$) | $k_{des}$ (s$^{-1}$) | $K$ ($k_{ads}$/$k_{des}$, ppm$^{-1}$) |
|---|---|---|---|
| $Cu_3(HHTP)_2$ powder | $8.77 \pm 0.26 \times 10^{-3}$ | $8.60 \pm 0.08 \times 10^{-5}$ | $10.2 \pm 0.3$ |
| $Cu_3(HHTP)_2$ thin-film | $1.51 \pm 0.03 \times 10^{-3}$ | $2.60 \pm 0.05 \times 10^{-5}$ | $58.2 \pm 1.2$ |
| Pt@$Cu_3(HHTP)_2$ thin-film | $2.34 \pm 0.22 \times 10^{-3}$ | $4.83 \pm 0.49 \times 10^{-5}$ | $48.5 \pm 4.6$ |

$k_{ads}$ is adsorption rate constant, $k_{des}$ is desorption rate constant, and $K$ is the equilibrium constant.

**Supplementary Table 8.** Summary of sensing properties of 2D material-based $NO_2$ sensors operated at room temperature in air.

| Materials | Measurement | Reponse definition | Humidity | Response | Detection limit | Ref.[a] |
|---|---|---|---|---|---|---|
| **Pt@$Cu_3$(HHTP)$_2$ thin-film** | Resistance | $\Delta R_g/R_0$ | Dry | 89.9% to 3 ppm | 0.1 ppm | **This work** |
| **$Cu_3$(HHTP)$_2$ thin-film** | Resistance | $\Delta R_g/R_0$ | Dry | 53.7% to 3 ppm | 0.1 ppm | **This work** |
| $Cu_3$(HHTP)$_2$ powder | Resistance | $\Delta R_g/R_a$ | Dry | 11.8% to 3 ppm | 1 ppm | **This work** |
| Pd@$Cu_3$(HHTP)$_2$ powder | Resistance | $\Delta R_g/R_a$ | Dry | 42.8% to 3 ppm | 1 ppm | 6 |
| MOF-templated PdO-$Co_3O_4$ | Resistance | $\Delta R_g/R_a$ | Dry | 5% to 5 ppm | 1 ppm | 7 |
| MOF-derived carbons | Resistance | $\Delta R_g/R_a$ | Dry | 1% to 5 ppm | 0.1 ppm | 8 |
| Scrolled graphene | Resistance | $\Delta R_g/R_a$ | Dry | 67.9% to 3 ppm | 1 ppm | 9 |
| Sulfonated RGO[b] | Resistance | $\Delta R_g/R_a$ | Dry | 58% to 10 ppm | 0.12 ppm | 10 |
| $In_2O_3$/RGO | Resistance | $\Delta R_g/R_a$ | Dry | 50% at 10 ppm | 1 ppm | 11 |
| $MoS_2$ nanosheets | Resistance | $\Delta R_g/R_a$ | Dry | 15% at 5 ppm | 1.5 ppm | 12 |
| $WS_2$/Ag nanowire | Resistance | $\Delta R_g/R_a$ | Dry | 58% at 25 ppm | 1 ppm | 13 |
| $WS_2$ nanosheets | Resistance | $\Delta R_g/R_a$ | Dry | 38 % at 2 ppm | 0.1 ppm | 14 |
| CdTe/$MoS_2$ | Resistance | $\Delta R_g/R_a$ | Dry | 27% at 5 ppm | 0.1 ppm | 15 |
| RGO/$MoS_2$ | Conductance | $\Delta G_g/G_a$ | Dry | 28% at 4 ppm | 1 ppm | 16 |

Ref.[a] is reference. RGO[b] is the reduced graphene oxide.

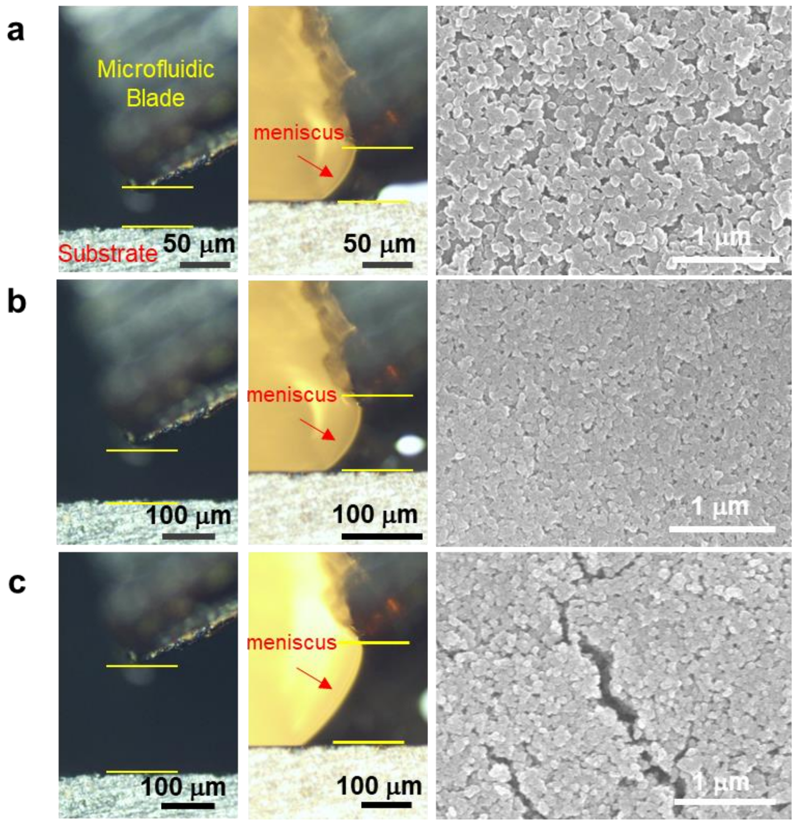


**Supplementary Fig. 1** Optical side-view images of the edge of the blade, and SEM images showing differences in film quality at various distances between the blade and the substrate: a) 50 µm, b) 100 µm, and c) 200 µm.

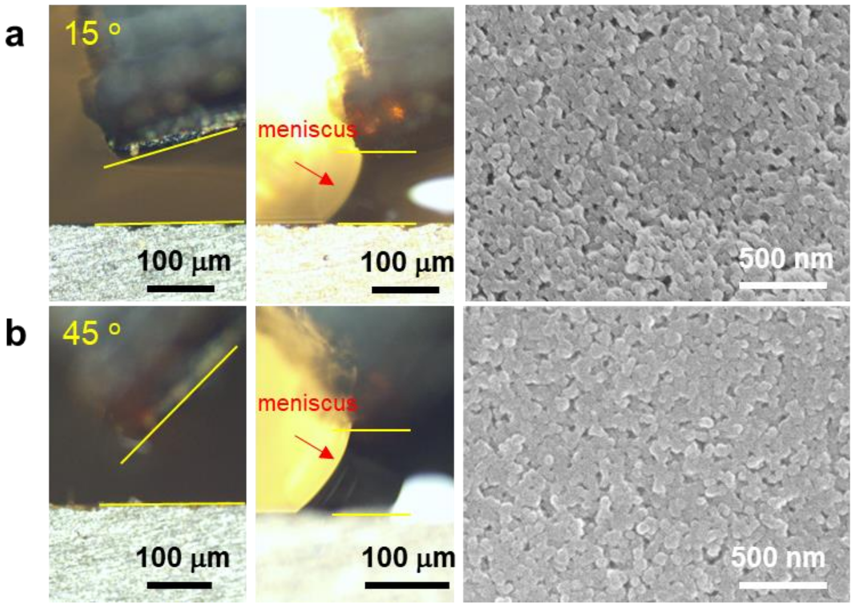


**Supplementary Fig. 2** Optical side-view images of the blade, and SEM images showing film quality at different blade angles: a) 15° and b) 45°. Similar looking films were attained at different angles.

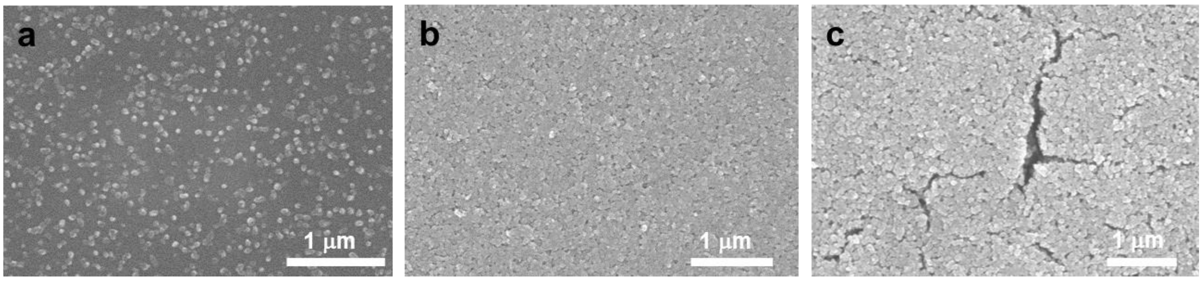


**Supplementary Fig. 3** SEM images showing the film quality of Pt@$Cu_3(HHTP)_2$ thin-film with different temperature: **(a)** 130 °C, **(b)** 150 °C, and **(c)** 160 °C.

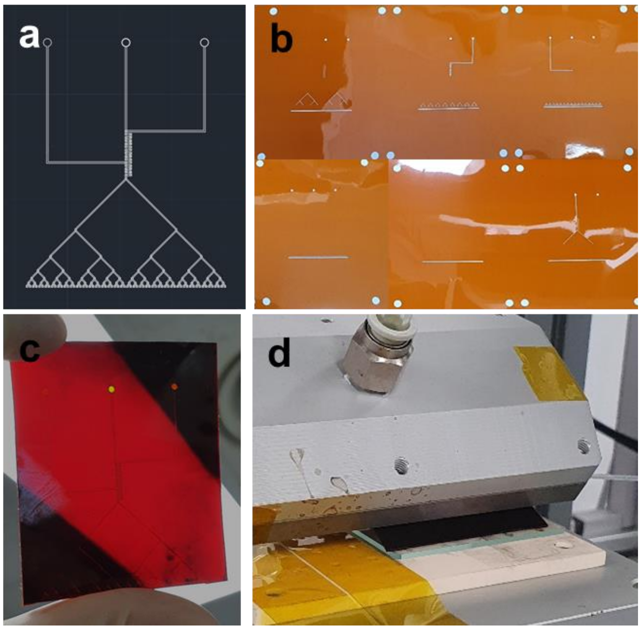


**Supplementary Fig. 4** Fabrication of microfluidic blade. **(a)** CAD image of the microfluidic blade channel design, **(b)** Photo image of laser-ablated polyimide film with microchannel design on each layer. **(c)** Photo image of fabricated microfluidic blade and **(d)** Photo image of solution shearing process with the fabricated microfluidic blade.

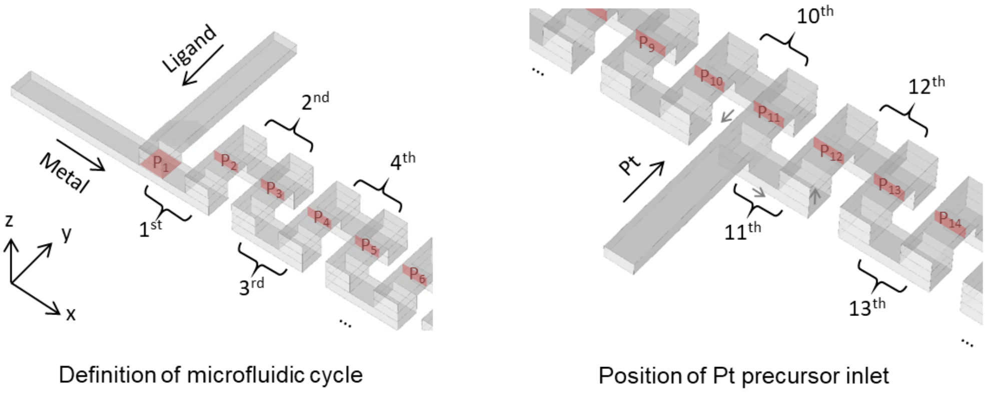


**Supplementary Fig. 5** Computational fluid dynamics simulation of microfluidic channels.

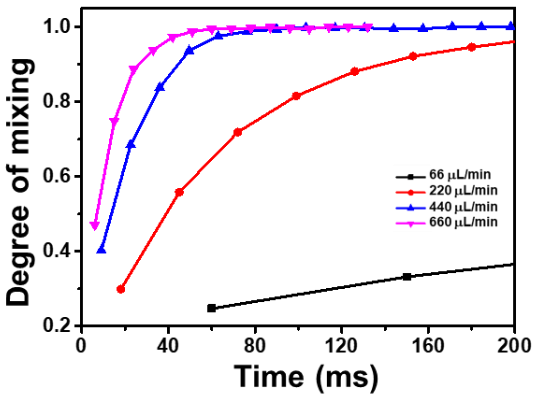


**Supplementary Fig. 6** Effect of mixing time on the degree of mixing at various flow rates.

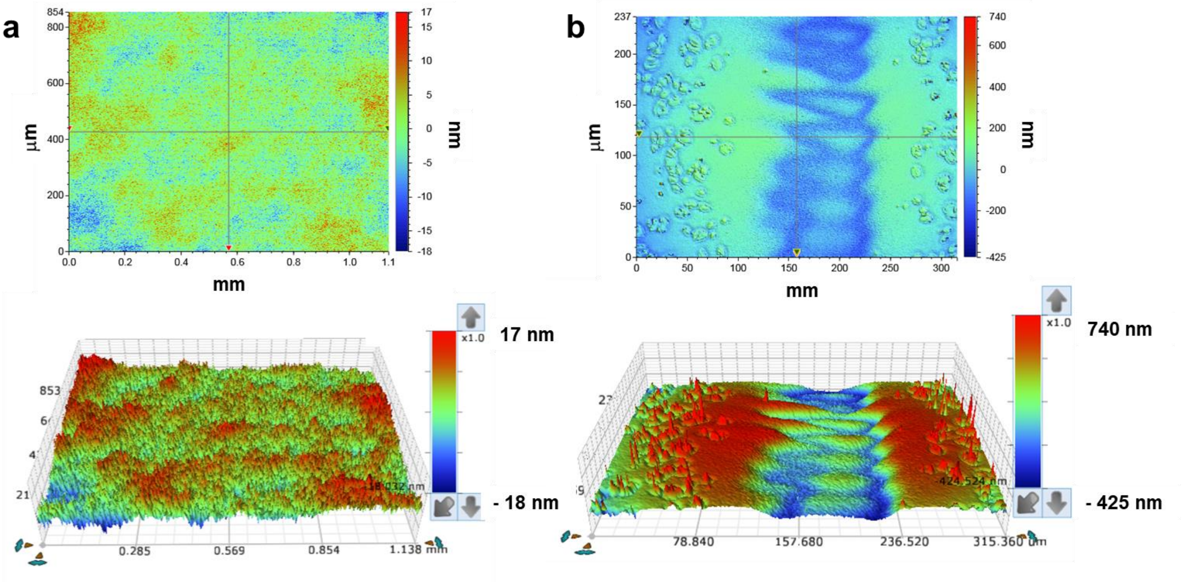


**Supplementary Fig. 7** Surface roughness profile measurement of Pt@$Cu_3(HHTP)_2$ thin-film fabricated by **(a)** MiCS and **(b)** conventional solution shearing at 150 °C (shearing speed: 5 mm/s). ($R_a$ values: **(a)** 2.8 nm, **(b)** 92.3 nm)

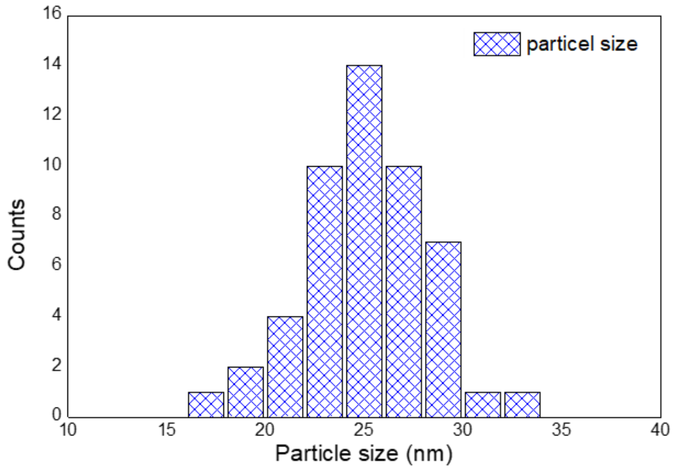


**Supplementary Fig. 8** Size distribution of Pt@$Cu_3(HHTP)_2$ crystallite in the thin-film. The crystallite size of Pt@$Cu_3(HHTP)_2$ was measured by using the high-resolution SEM analysis (**Fig. 2h**).

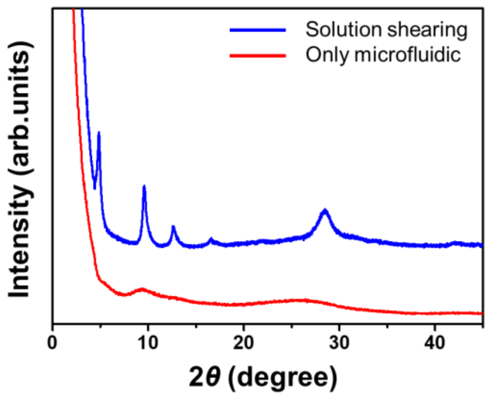


**Supplementary Fig. 9** XRD patterns of Pt@$Cu_3(HHTP)_2$ after MiCS process, and only after microfluidic synthesis process without solution shearing.

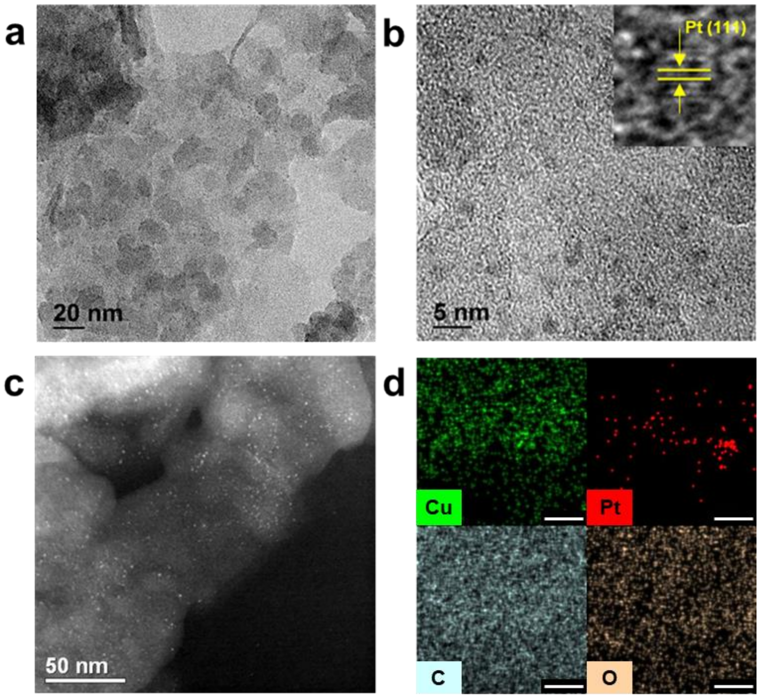


**Supplementary Fig. 10 (a)** TEM image of Pt@$Cu_3(HHTP)_2$, **(b)** HRTEM image of Pt@$Cu_3(HHTP)_2$, **(c)** STEM image of Pt@$Cu_3(HHTP)_2$ and **(d)** TEM-EDS mapping of Pt@$Cu_3(HHTP)_2$. Scale bars in (d) are 5 nm.

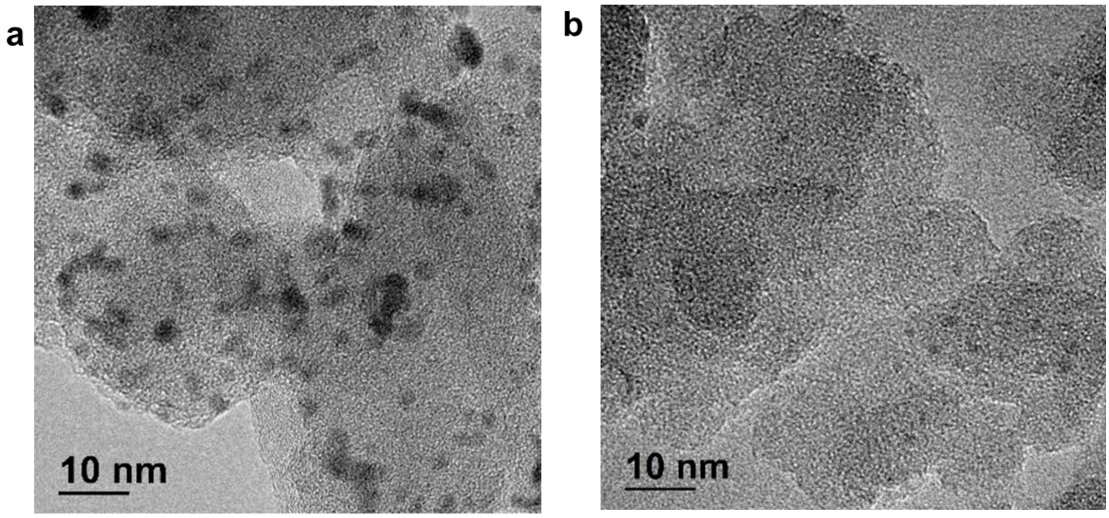


**Supplementary Fig. 11** HRTEM images of Pt@$Cu_3(HHTP)_2$ under different synthesis processes.: **(a)** synthesized using bulk synthetic technique, **(b)** synthesized using microfluidic-based solution shearing process.

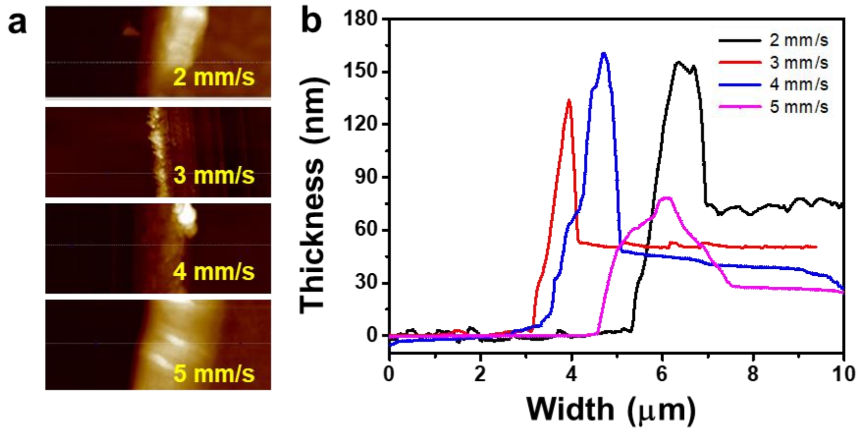


**Supplementary Fig. 12 (a)** AFM topographic images and **(b)** thickness profile of a manually scratched Pt@$Cu_3(HHTP)_2$ films at various shearing speeds.

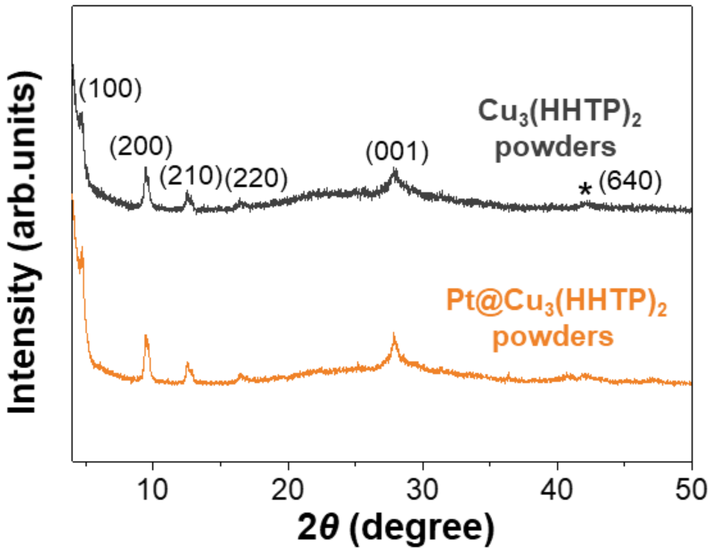


**Supplementary Fig. 13** XRD analysis of bulk grown $Cu_3(HHTP)_2$ powders and $Pt@Cu_3(HHTP)_2$ powders.

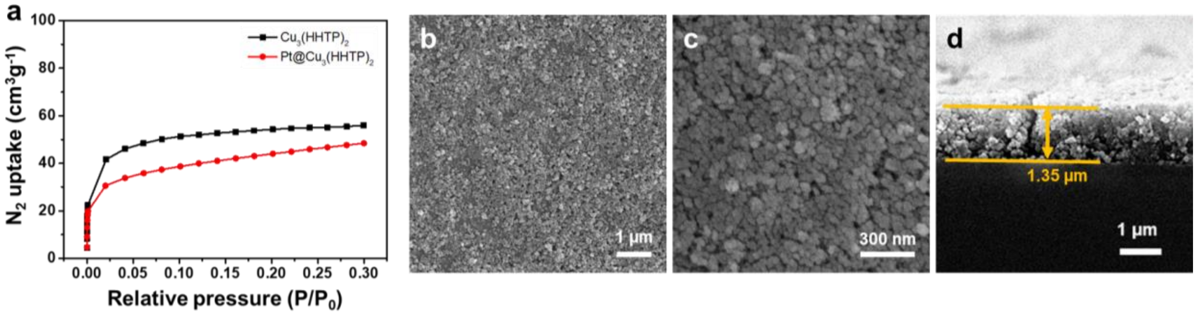


**Supplementary Fig. 14 (a)** $N_2$ adsorption and desorption isotherms of the $Cu_3(HHTP)_2$ and $Pt@Cu_3(HHTP)_2$ samples at 77 K. **(b–d)** SEM images of the thick sample for BET analysis: top view images under **(b)** low and **(c)** high magnification, and **(d)** cross-sectional image. It is noted that the crack in the middle of the sample in **(d)** was generated during the sampling process of the cross-section. The thickness of the thick films was about 1.35 μm. Thus, in order to collect 20 mg of the samples for BET analysis, we fabricated 15–20 thick films (shearing size: about 45 × 60 mm).

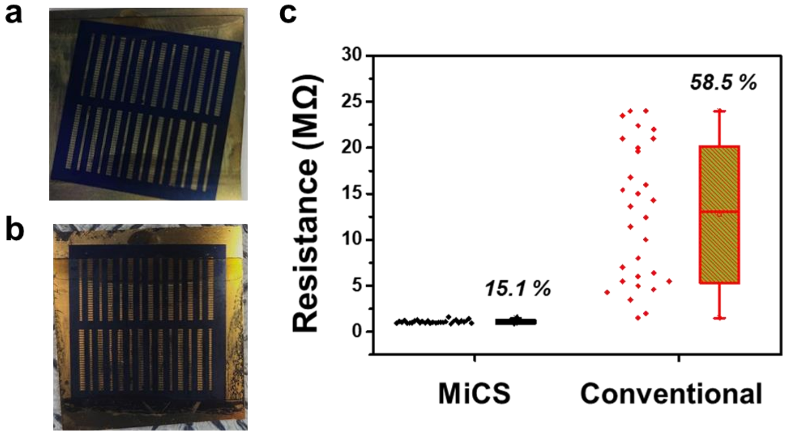


Supplementary Fig. 15 Comparison of electrical properties of C-MOF films fabricated through MiCS process and conventional solution shearing process: Optical images of C-MOF films fabricated by (a) MiCS process and (b) conventional solution shearing process. (c) Comparison of electrical properties of C-MOF films fabricated using MiCS process and conventional solution shearing process. Coefficient of variation (%) for each process is italicized. The error bars present the standard deviation of the samples ($N \geq 30$).

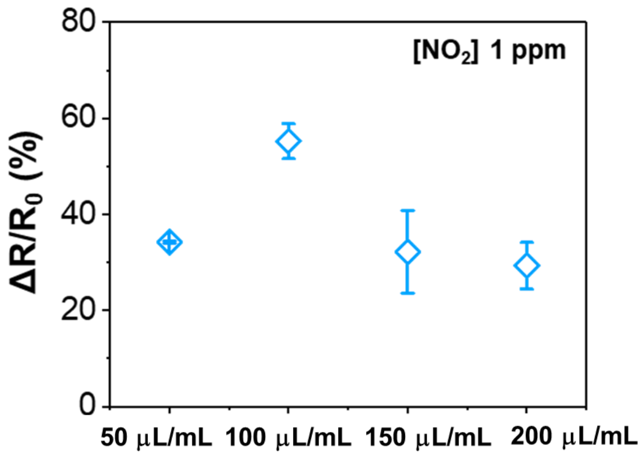


**Supplementary Fig. 16** Responses of Pt@$Cu_3(HHTP)_2$ thin-film to different flow rates of Pt precursor solution. The error bars present the standard deviation of the sensors ($N \geq 4$).

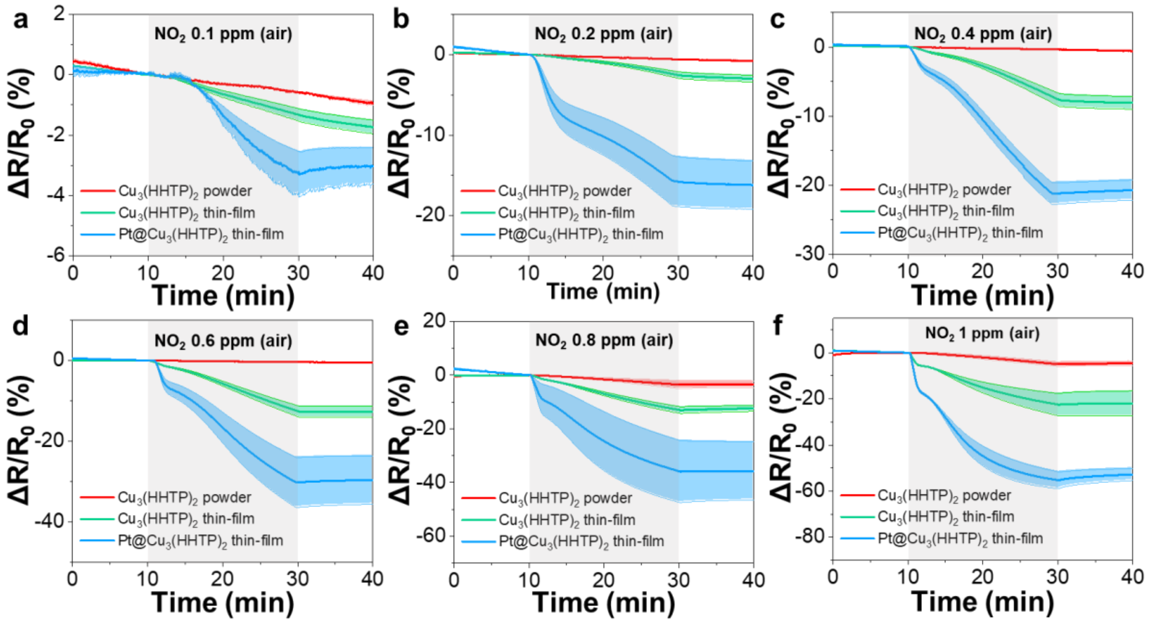


**Supplementary Fig. 17** Response traces of the sensors to 0.1–1 ppm of $NO_2$: **(a)** 0.1, **(b)** 0.2, **(c)** 0.4, **(d)** 0.6, **(e)** 0.8, and **(f)** 1 ppm of $NO_2$. The shaded area indicates the standard deviation of the sensors (N $\geq$ 4).

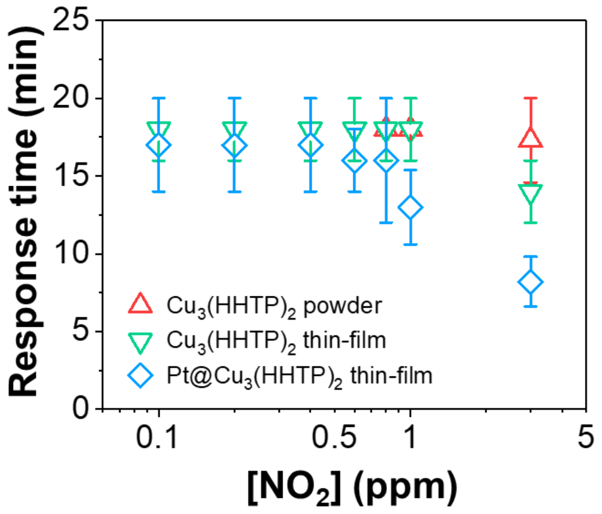


**Supplementary Fig. 18** Response times of the sensors to 0.1–3 ppm of $NO_2$. The error bars present the standard deviation of the sensors ($N \geq 4$).

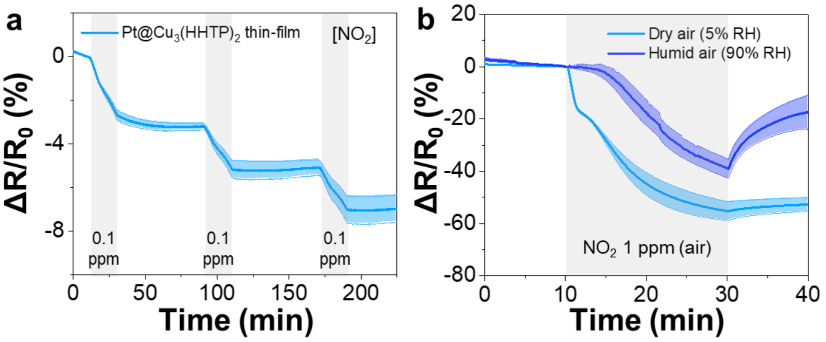


**Supplementary Fig. 19** Additional sensing results. **(a)** Response traces of Pt@$Cu_3(HHTP)_2$ thin-film to multiple exposures of $NO_2$ 0.1 ppm. **(b)** Response traces of the sensors to $NO_2$ 1 ppm in dry and humid air. The shaded area in each graph presents the standard deviation of the sensors ($N \geq 4$).

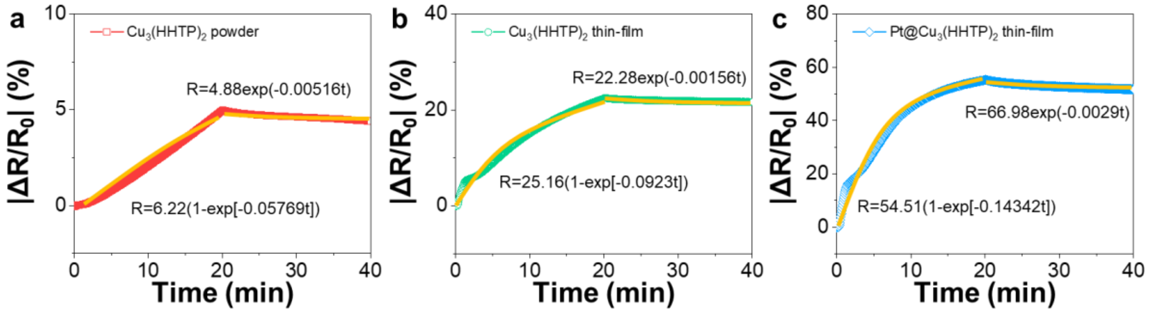


**Supplementary Fig. 20** Exponential fitting curves of the sensors. Response traces of the sensors to 1 ppm of $NO_2$: **(a)** $Cu_3(HHTP)_2$ powder, **(b)** $Cu_3(HHTP)_2$ thin-film, and **(c)** Pt@$Cu_3(HHTP)_2$ thin-film. Reaction rate constants were calculated by the exponential fitting of response traces.

In detail, $NO_2$ reaction rate constants of the sensors were calculated by (1) the mass action law of $NO_2$ reactions and (2) the assumption that response is proportional to the amounts of adsorbed $NO_2$ molecules[2,3]. The reaction rate constants were obtained from the exponential fitting of the response trace curves [*R(t)*] using following equations.

$$R(t) \text{ for } NO_2 \text{ adsorption} = R_{max} \cdot \frac{C_g K}{1+C_g K}\left(1 - \exp\left[-\frac{1+C_g K}{K} \cdot k_{ads} \cdot t\right]\right) \quad (1)$$

$$R(t) \text{ for } NO_2 \text{ desorption} = R_0 \exp[-k_{des} \cdot t] \quad (2)$$

where $K$ is the equilibrium constant, $k_{ads}$ is the adsorption rate constant, and $K_{des}$ is the desorption rate constant, $R_{max}$ is the maximum response, and $C_g$ is the concentration of gas ($NO_2$) molecules.

## Supplementary References


1. Ko, M., Mendecki, L., Mirica. K.A. Conductive two-dimensional metal-organic frameworks as multifunctional materials. *Chem. Commun.* **54**, 7873-7891 (2018).

2. Campbell, M.G., Sheberla, D., Liu, S.F., Swager, T.M., Dinca, M. $Cu_3$(hexaiminotriphenylene$_2$: an electrically conductive 2D metal-organic framework for chemiresistive sensing. *Angew. Chem. Int. Ed.* **54**, 4349-4352 (2015).

3. Mendecki, L., Mirica, K.A. Conductive metal-organic frameworks as ion-to-electron transducers in potentiometric sensors. *ACS Appl. Mater. Interfaces* **10**, 19248-19257 (2018).

4. Yao, M.S. *et al.* Layer-by-layer assembled conductive metal–organic framework nanofilms for room-temperature chemiresistive sensing. *Angew. Chem., Int. Ed.* **56**, 16510-16514 (2017).

5. Chen, I.-F., Lu, C.-F., Su, W.-F. Highly conductive 2D metal–organic framework thin film fabricated by liquid–liquid interfacial reaction using one-pot-synthesized benzenehexathiol. *Langmuir* **34**, 15754-15762 (2018).

6. Koo, W.-T., Kim, S.-J., Jang, J.-S., Kim, D.-H., Kim, I.-D. Catalytic metal nanoparticles embedded in conductive metal–organic frameworks for chemiresistors: highly active and conductive porous materials. *Adv. Sci.* **6**, 1900250 (2019).

7. Choi, S.-J., Choi, H.-J., Koo, W.-T., Huh, D., Lee, H., Kim, I.-D. Metal-organic framework-templated PdO-$Co_3O_4$ nanocubes functionalized by SWCNTs: improved $NO_2$ reaction kinetics on flexible heating film. *ACS Appl. Mater. Interfaces* **9**, 40593-40603 (2017).

8. Rui, K. *et al.* Dual-function metal-oganic framework-based wearable fibers for gas probing and energy storage. *ACS Appl. Mater. Interfaces* **10**, 2837-2842 (2018).

9. Chen, Z. *et al.* Mimicking a dog's nose: scrolling graphene nanosheets. *ACS Nano* **12**, 2521-2530 (2018).

10. Yuan, W., Liu, A., Huang, L., Li, C., Shi, G. High-performance $NO_2$ sensors based on chemically modified graphene. *Adv. Mater.* **25**, 766-771 (2013).

11. Yang, W., Wan, P., Zhou, X., Hu, J., Guan, Y., Feng, L. Additive-free synthesis of $In_2O_3$ cubes embedded into graphene sheets and their enhanced $NO_2$ sensing performance at room temperature. *ACS Appl. Mater. Interfaces* **6**, 21093-21100 (2014).

12. Cho, B. *et al.* Charge-transfer-based gas sensing using atomic-layer $MoS_2$. *Sci. Rep.* **5**, 8052 (2015).

13. Ko, K.Y. *et al.* Improvement of gas-sensing performance of large-area tungsten disulfide nanosheets by surface functionalization. *ACS Nano* **10**, 9287-9296 (2016).

14. Xu, T. *et al.* The ultra-high $NO_2$ response of ultra-thin $WS_2$ nanosheets synthesized by hydrothermal and calcination processes. *Sens. Actuators, B* **259**, 789-796 (2018).

15. Jaiswal, J., Sanger, A., Tiwari, P., Chandra, R. $MoS_2$ hybrid heterostructure thin film decorated with CdTe quantum dots for room temperature $NO_2$ gas sensor. *Sens. Actuators, B* **305**, 127437 (2020).

16. Zhou, Y., Liu, G., Zhu, X., Guo, Y. Ultrasensitive $NO_2$ gas sensing based on rGO/$MoS_2$ nanocomposite film at low temperature. *Sens. Actuators, B* **251**, 280-290 (2017).
